# In Vivo Base Editing Partially Rescues Bone Dysplasia in a Mouse Model of Hutchinson‐Gilford Progeria Syndrome

**DOI:** 10.1111/acel.70700

**Published:** 2026-09-03

**Authors:** Wayne A. Cabral, Caleb M. Grenko, Diana Yeritsyan, Indeevar Beeram, Shubham Laiwala, Lukas R. Johnson, Urraca L. Tavarez, Tingfen Yan, Stephen M. Wincovitch, Luke W. Koblan, Xin D. Gao, Meirui An, Narisu Narisu, Leslie G. Biesecker, David R. Liu, Ara Nazarian, Francis S. Collins, Michael R. Erdos

**Affiliations:** ^1^ Molecular Genetics Section, Center for Precision Health Research National Human Genome Research Institute, NIH Bethesda Maryland USA; ^2^ Skeletal Mechanobiology Laboratory National Institute of Arthritis and Musculoskeletal and Skin Diseases, NIH Bethesda Maryland USA; ^3^ Musculoskeletal Translational Innovation Initiative, Carl J. Shapiro Department of Orthopedic Surgery Beth Israel Deaconess Medical Center and Harvard Medical School Boston Massachusetts USA; ^4^ Advanced Imaging and Analysis Core Facility National Human Genome Research Institute, NIH Bethesda Maryland USA; ^5^ Merkin Institute of Transformative Technologies in Healthcare Broad Institute of Harvard and MIT Cambridge Massachusetts USA; ^6^ Department of Chemistry and Chemical Biology Harvard University Cambridge Massachusetts USA; ^7^ Howard Hughes Medical Institute Harvard University Cambridge Massachusetts USA; ^8^ Department of Ophthalmology University of Pittsburgh School of Medicine Pittsburgh Pennsylvania USA; ^9^ The Progeria Research Foundation Peabody Massachusetts USA

**Keywords:** adenine base editor, bone dysplasia, Hutchinson‐Gilford progeria syndrome, lamin A/C

## Abstract

Hutchinson‐Gilford progeria syndrome (HGPS) is a premature aging disorder affecting tissues of mesenchymal origin. Most patients harbor a c.1824C>T/p.G608= variant, commonly described as G608G, in exon 11 of *LMNA* that leads to aberrant splicing and production of the toxic progerin protein. In addition to cardiovascular, dermal, and adipose tissue deterioration, HGPS mouse models also develop progressive bone dysplasia that occurs in patients. Here we characterize the efficacy of in vivo mutation correction with an adenine base editor (ABE) to rescue structural and functional defects in HGPS transgenic murine bone tissue. Treatment of double‐copy transgenic osteoblast cultures with a lentiviral‐delivered CRISPR‐Cas9 ABE achieved nearly 40% gene correction in vitro, resulting in significant reduction of progerin transcripts and protein, in the absence of selective agents. Furthermore, gene correction improved progeroid osteoblasts' capacity to deposit and mineralize extracellular matrix compared to untreated cultures. In vivo, a single intravenous dose of AAV9‐delivered ABE corrected the mutation, achieving ~14%, ~22%, ~10% and < 1% correction in bone by six months of age when administered at P3, P14, 1 and 4 months of age, respectively. Partially rescued bone structural and physical parameters were observed in P14‐treated mice with concomitant normalization of gene transcriptional programs and intracellular signaling pathways involved in bone remodeling. This work demonstrates in vivo delivery of a locus‐specific DNA base editor to bone tissue, delineates the timing of treatment required for maximum efficacy, and suggests that this system might be tailored for application to other monogenic bone disorders.

## Graphical abstract

Hutchinson‐Gilford progeria syndrome (HGPS) is a premature aging disorder caused by a mutation in *LMNA* that produces the toxic progerin protein. In this study, an adenine base editor delivered via AAV9 achieved partial gene correction in HGPS mice when given at two weeks of age, partially rescuing bone structural and gene expression parameters and suggesting potential application to other monogenic bone disorders.

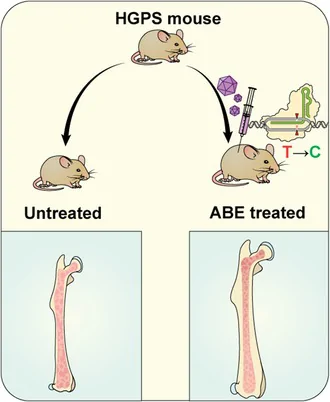

## 1 Introduction

Hutchinson‐Gilford progeria syndrome (HGPS) is a rare segmental premature‐aging disorder most commonly caused by a specific de novo mutation in the *LMNA* gene, encoding lamin A, a major constituent of the nuclear lamina. The classic HGPS mutation (c.1824C>T, p.G608=, commonly referred to as G608G) activates a cryptic splice site in exon 11 resulting in a truncated form of lamin A lacking an internal cleavage recognition site required for appropriate proteolytic processing (De Sandre‐Giovannoli et al. 2003; Eriksson et al. 2003). This permanently farnesylated protein, termed progerin, functions in a dominant‐negative manner by becoming anchored to the nuclear membrane, where it disrupts the normal functions of the nuclear lamina. At the cellular level, progerin expression induces nuclear morphological defects, altered heterochromatin structure, defects in DNA repair, dysregulated gene expression, genomic instability, and premature senescence (Batista et al. 2023).

Although individuals with HGPS have a normal appearance at birth, they typically develop failure to thrive, lipodystrophy, scleroderma‐like changes to the skin, and alopecia within their first year of life. Patients with HGPS also develop a bone dysplasia characterized by skeletal manifestations that include an abnormally shaped rib cage with thin, tapered ribs, dental crowding, acro‐osteolysis and clavicular resorption, and gracile long bones with metaphyseal demineralization (Gordon et al. 2011). The skeletal phenotype can also include hip and skull abnormalities, especially coxa valga with occasional hip subluxation, diastasis of the cranial sutures, and Wormian bones. It is, however, the progressive cardiovascular disease resembling atherosclerosis that is of greatest threat for these patients, which without treatment leads to death at 13 to 14 years of age due to heart attacks and strokes (Gordon et al. 2007).

The generation of a humanized transgenic mouse harboring the *LMNA* G608G variant within a 164‐kb bacterial artificial chromosome (BAC), designated here as *LMNA* ^G/+^, provides a useful tool for testing novel therapeutic approaches in the context of the human gene, transcript and protein sequence (Varga et al. 2006). Although single‐copy transgenic mice lack the phenotypic features of HGPS outside of the vascular system, double‐copy mice (*LMNA* ^G/G^) faithfully replicate the pathologic changes that occur in the human disorder, including severe vascular smooth muscle cell (VSMC) loss with adventitial thickening of large vessels, loss of subcutaneous adipose tissue and progressive bone deterioration (Cabral et al. 2021). We recently demonstrated that single intravenous treatment with a seventh generation CRISPR‐Cas9 adenine base editing system (ABE) achieved partial correction of the *LMNA* transgene across multiple tissues and significantly extended the lifespan of this mouse model of HGPS (Koblan et al. 2021). Notably, we found that treatment of transgenic mice at post‐natal days 3 and 14 resulted in correction of 14%–22% of transgene copies in bone tissue by six months of age, thereby creating a somatic mosaic model for further characterization.

These findings prompted investigation into whether partial correction of *LMNA* in skeletal tissues can meaningfully modify bone pathology in HGPS, an especially relevant question given the evidence from monogenic skeletal disorders demonstrating that disease severity may be strongly influenced by the extent and cellular distribution of pathogenic variants. While activating mutations of intracellular signaling components can induce progressive skeletal dysplasia, a decreased burden of dominant‐negative or loss‐of‐function mutations in genes encoding extracellular matrix structural and signaling proteins within bone cell populations is compatible with less severe defects in skeletal growth and mineral density (Boyce and Collins 2020; Cabral and Marini 2004; Desir et al. 2012; Geister and Camper 2015; Hyland et al. 2003; Kang et al. 2018, 2020; Lim et al. 2014; Lindhurst et al. 2011; Muurinen et al. 2022). We hypothesized that in vivo gene editing of *LMNA*, resulting in partial correction, would be sufficient in whole or in part to rescue the progressive bone phenotype that develops throughout the lifespan of HGPS transgenic mice, and potentially in the future for patients with HGPS. Here we show partial restoration of bone structural and physical properties, and tissue‐level osteogenic gene expression programs, following treatment with a targeted DNA base editing system in a humanized transgenic mouse model of HGPS.

## 2 Results

**FIGURE 1:**
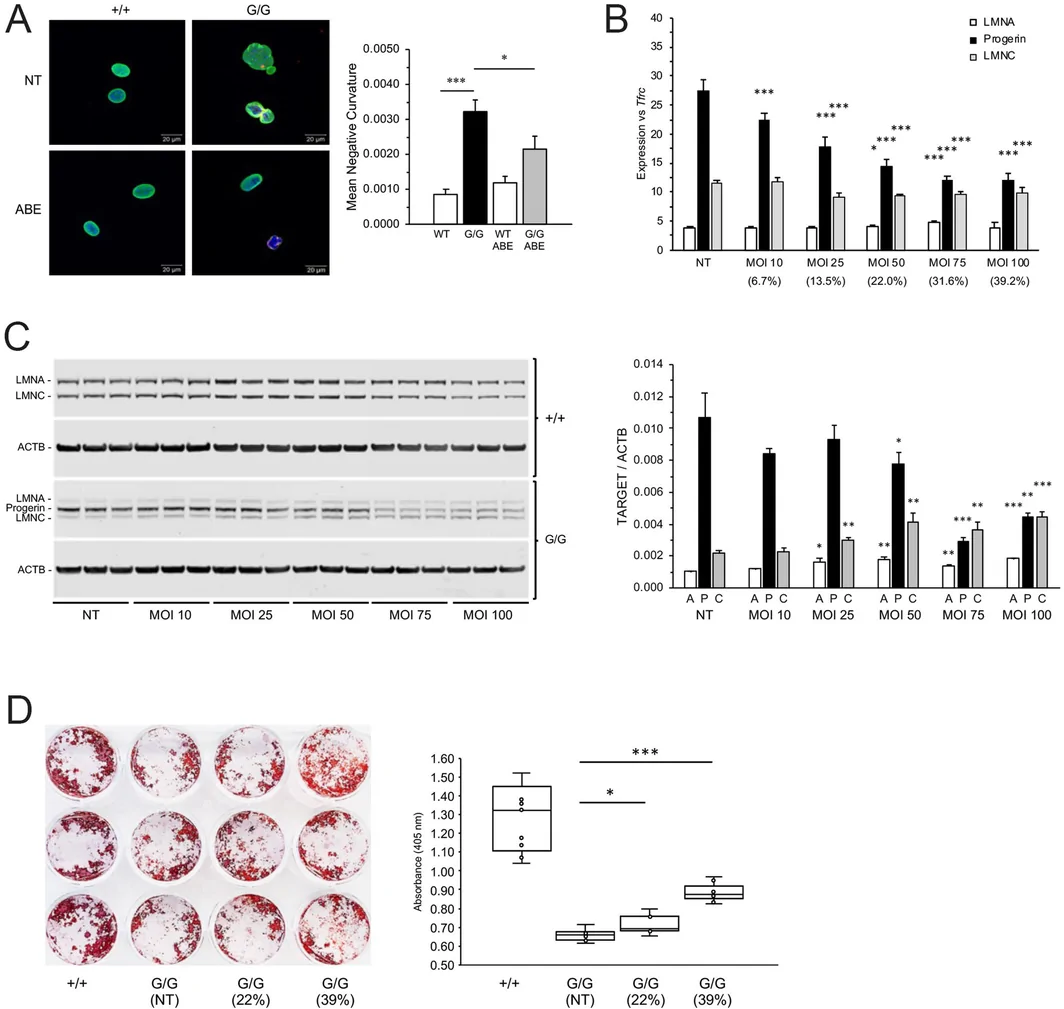
Adenine base editing system corrects the *LMNA* c.1824C>T mutation and improves functioning of murine HGPS osteoblasts in vitro. (A) Left, nuclear blebbing, a hallmark feature of HGPS cells, is observed in non‐treated (NT) calvarial osteoblasts derived from *LMNA* ^G/G^ newborn mice. Note the presence of a corrected and uncorrected cell in the ABE‐treated *LMNA* ^G/G^ confocal image. Right, treatment with ABE partially restores nuclear shape, measured as mean negative curvature, in *LMNA* ^G/G^ osteoblasts (G/G) relative to wild‐type cells (WT). *N* = 84–200 nuclei per treatment group. (B) Quantitative RT‐PCR analyses of transgene‐derived *LMNA* transcripts relative to *Tfrc* reference transcripts at increasing ABE multiplicities of infection (MOI). The fraction of corrected transgenes is noted in parentheses. (C) Left, immunoblots of cell lysates from wild‐type (+/+) and double‐copy transgenic (G/G) cultured osteoblasts treated with ABE in vitro. Quantitation of transgene‐derived lamin A (A), progerin (P) and lamin C (C), relative to beta‐Actin (ACTB), proteins is shown at right. (D) In vitro mineralization assay shows improvement in osteoblast deposition of extracellular matrix and hydroxyapatite. Left, cultures stained with alizarin red following ABE treatment. Right, quantitation of extracted stain using an absorbance‐based assay. *p < 0.05; **p < 0.01; ***p < 0.001.

Preliminary in vitro experiments were performed to confirm that treatment with an ABE could edit and elicit functional improvement of murine‐derived osteoblasts. Double‐copy transgenic (*LMNA* ^G/G^) osteoblast cultures were transduced with lentiviral‐delivered ABE, grown for an additional seven days, and then collected for further analysis. Although ABE expression should confer puromycin resistance in transduced cells, we avoided selection to replicate in vivo treatment conditions. Biological replicate cultures were treated over a range of Multiplicities of Infection (MOIs) to correlate the extent of mutation editing with transcript levels, protein levels, and nuclear morphological changes. These experiments indicated that culturing of *LMNA* ^G/G^ osteoblasts with ABE‐expressing lentivirus was sufficient to reduce the appearance of intracellular progerin and induce observable changes to nuclear shape in these cells (Figure S1, Figure 1A). We thus proceeded with a detailed characterization of cellular alterations induced by ABE‐catalyzed transgene correction.

Droplet Digital PCR (ddPCR) allele variant analysis demonstrated c.1824T>C correction in 6.7% ± 1.3% to 39.2% ± 1.0% of transgene copies from the range of MOIs used with this viral preparation (Figure S2A). The extent of transgene correction in vitro corresponded with a 7.1% ± 3.7% to 56.0% ± 4.9% reduction in progerin transcripts with no detectable increase in normal *LMNA* expression compared to untreated cells (Figure 1B, Figure S2B). Progerin protein abundance decreased as much as 72.6% ± 2.1% with a corresponding increase in full‐length LMNA of up to 73.0% ± 1.2% (Figure 1C, Figure S2C). Assessment of functional rescue in treated cultures was performed using in vitro mineralization assays, which revealed that even minimal levels of transgene correction in osteoblasts were sufficient to significantly improve their ability to deposit and mineralize extracellular matrix. Following three weeks of osteogenic stimulation, untreated *LMNA* ^G/G^ cultures contained 49% ± 12% less mineral than wild‐type control cultures (*p* < 0.02), whereas ABE‐treated *LMNA* ^G/G^ cells achieved a 9% ± 3% (*p* < 0.05) to 37% ± 4% (*p* < 0.001) increase of deposited mineral, but did not attain wild‐type values (Figure 1D). These results, however, confirmed that single treatment with ABE is sufficient to reduce progerin production and improve the function of *LMNA* ^G/G^ osteoblasts in vitro.

Our in vivo approach for delivering ABE to multiple mouse tissues utilized AAV9 capsid, chosen for its broad tissue tropism, clinical validation, and ability to transduce progeria‐relevant tissues (Koblan et al. 2021). Due to the size of the vector required to encode ABE and the limitations of AAV9, this approach depended on trans‐splicing inteins to reconstitute the full‐length base editor in cells from a pair of AAVs, each expressing one half of the base editor, and adapted to deliver ABEmax‐VRQR and the *LMNA* c.1824‐targeting sgRNA (Koblan et al. 2021; Levy et al. 2020; Villiger et al. 2018). We found that a single injection of ABE‐encoding AAVs resulted in modest to high levels of correction (10%–60%) of the causative *LMNA* point mutation in various organs of *LMNA* ^G/G^ mice (Koblan et al. 2021). Specifically, mice given retro‐orbital injections at 3 (P3) and 14 (P14) days after birth contained 3.5% ± 1.4% and 11.1% ± 3.2% of transgene copies, respectively, with the c.1824T>C edit in tibial bone tissue at six weeks of age (Koblan et al. 2021). By six months, however, P3‐ and P14‐treated mice had 13.9% ± 3.5% and 22.2% ± 9.3% of transgene copies corrected, respectively. In a separate experiment, treatment of mice at one and four months resulted in 9.9% ± 6.3% and 0.6% ± 0.3% of transgene copies that were corrected by six months (Table S1) suggesting that editing efficiency in bone tissue is maximized in our mouse model when treatment occurs at two weeks of age (P14). We therefore directed subsequent analyses on P3‐ and P14‐treated animals that demonstrated the greatest extent of editing.

**FIGURE 2:**
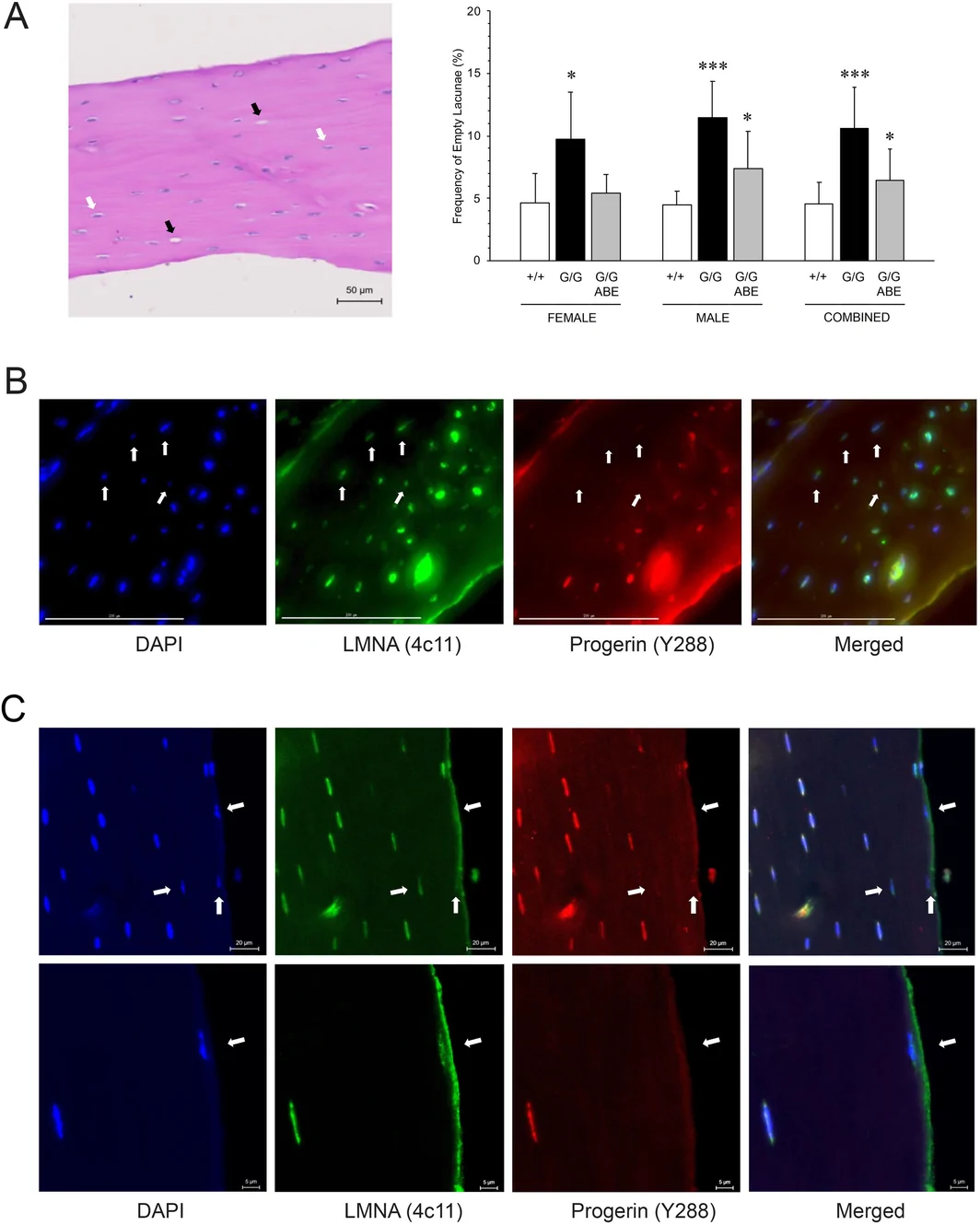
Edited cells localize to scattered clusters near bone tissue surfaces. (A) Left, representative H&E‐stained femora from *LMNA* ^G/G^ mouse illustrates cortical osteocyte lacunar occupancy. Both empty (black arrows) and occupied (white arrows) lacunae are identified by the absence or presence of nuclei; scale bar = 50 μm. Right, quantitation of lacunar occupancy. *N* = 12 (six males, six females) for wild‐type (WT), P14 saline‐treated *LMNA* ^G/G^ (Saline) and P14 ABE‐treated *LMNA* ^G/G^ (ABE) mice at six months of age; *p < 0.05; **p < 0.01; ***p < 0.001. (B, C) Representative confocal microscopic images of P14 ABE‐treated HGPS mouse femora at 6 months of age. Arrows indicate cells that are negative for progerin‐specific antibody (YZ288) reactivity, but positive for DAPI, normal mouse and human A‐type lamins (4c11) in (B) metaphyseal trabecular bone; 40× magnification; scale bar = 100 μm, and (C) diaphyseal cortical bone tissue. Top, 40× magnification; scale bar = 20 μm; Bottom, 60× magnification; scale bar = 5 μm.

Histologic evaluation of *LMNA* ^G/G^ femora in six‐month‐old mice initially suggested a nominal response to treatment. Upon hematoxylin and eosin (H&E) staining, we observed no significant differences in lacunar density among wild‐type (0.72 ± 0.13/μm^2^), saline‐treated (0.71 ± 0.10/μm^2^) and P14 ABE‐treated (0.70 ± 0.10/μm^2^) *LMNA* ^G/G^ mice, respectively, per diaphyseal femoral region that was analyzed. There was, however, a greater than two‐fold increase in the proportion of lacunae that were empty in saline‐treated *LMNA* ^G/G^ bone compared to wild‐type mice (*p* < 0.001). This fraction of empty lacunae was decreased 40% ± 21% in ABE‐treated versus saline‐treated *LMNA* ^G/G^ mice but remained statistically greater than wild‐type littermates (*p* = 0.04) (Figure 2A). More intense Safranin‐O staining of femoral distal growth plates revealed increased proteoglycan content within the hypertrophic region, with no obvious differences in growth plate cell number or width in saline‐ and ABE‐treated HGPS mice compared to wild‐type (Figures S3 and S4). Moreover, there was a near‐total absence of the cartilage‐to‐bone transitional zone in *LMNA* ^G/G^ mice, as observed in wild‐type mice, where Safranin‐O positive “spicules” protruded from the growth plate into the ossified trabecular tissue of the metaphyses. Rather, ossified tissue within this region of the primary spongiosa was reduced in thickness, as previously reported in the *Lmna* ^G609G/G609G^ mouse model (Blouin et al. 2024), and was not altered by ABE treatment.

**FIGURE 3:**
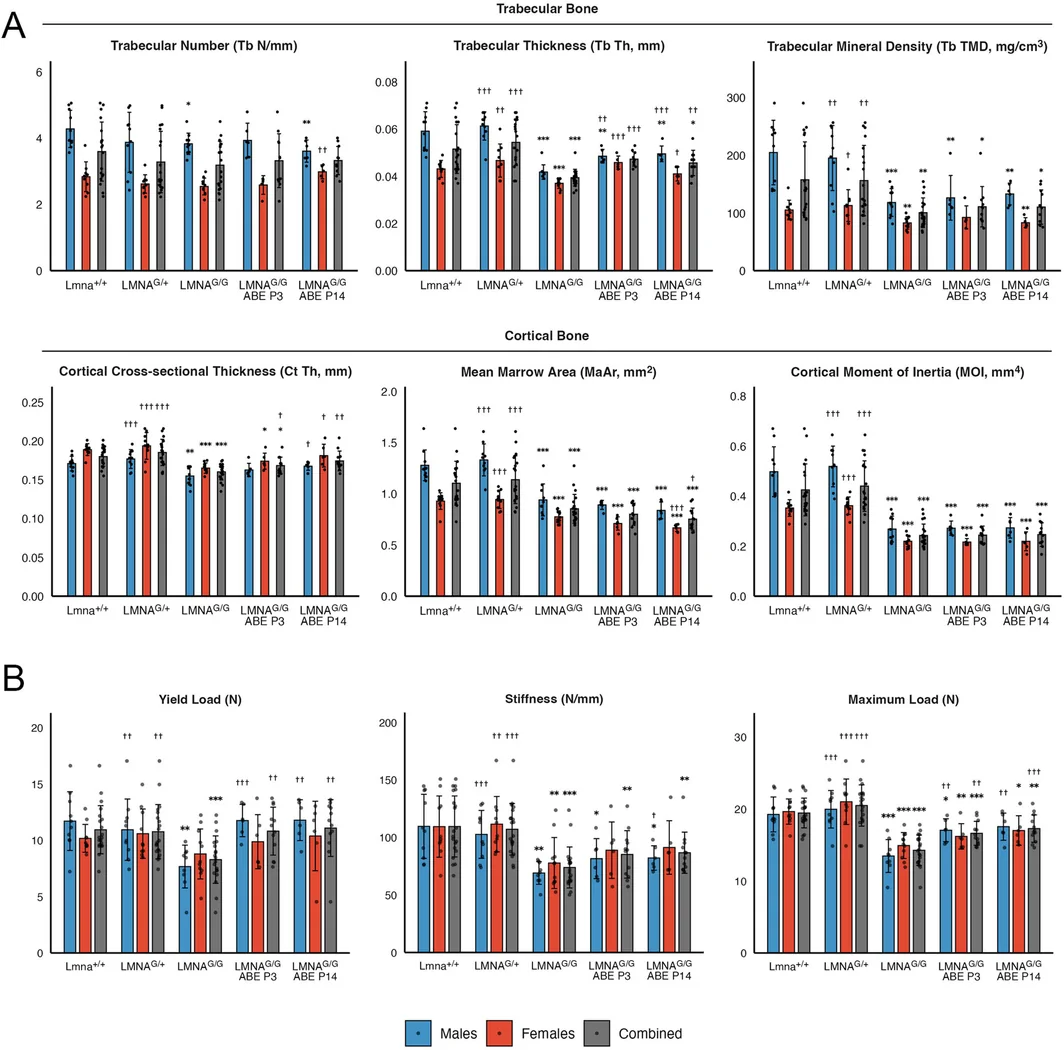
Femoral bone structural and physical parameters are partially rescued in ABE‐treated mice. (A) Trabecular and cortical structural parameters indicate smaller and structurally compromised femora of six month‐old *LMNA* ^G/G^ mice. ABE treatment at P3 and P14 produced partial structural improvements, including significant increases in trabecular and cortical thickness, particularly in P14‐treated mice. (B) Three‐point bending tests showed reduced mechanical properties of *LMNA* ^G/G^ femora at six months of age. Although ABE treatment at P3 and P14 partially improved mechanical parameters, particularly yield load and maximum load, stiffness was not fully recovered. *N* = 19 (10 males, nine females) wild‐type (*Lmna* ^+/+^), 19 (10 males, nine females) single‐copy (*LMNA* ^G/+^), 20 (10 males, 10 females) double‐copy (*LMNA* ^G/G^), 12 (six males, six females) P3‐treated double‐copy (*LMNA* ^G/G^ ABE P3) and 12 (six males, six females) P14‐treated double‐copy (*LMNA* ^G/G^ ABE P14) mice at six months of age. *p < 0.05; **p < 0.01; ***p < 0.001 versus *Lmna* ^+/+^ littermates; ^†^ *p* < 0.05, ^††^ *p* < 0.01, ^†††^ *p* < 0.001 versus untreated *LMNA* ^G/G^ littermates.

To determine what effects transgene editing had on bone structural and physical parameters in *LMNA* ^G/G^ mice, micro‐computed tomography (μCT) and mechanical testing were performed on femora collected from P3‐ and P14‐treated mice at six months of age and compared to age‐matched wild‐type, single‐copy transgenic (*LMNA* ^G/+^) and saline‐treated double‐copy transgenic littermates (Tables S2 and S3). Both trabecular and cortical structural parameters in the long bones of *LMNA* ^G/+^ mice were comparable to those of wild‐type (*Lmna* ^+/+^) littermates, indicating the absence of detectable skeletal abnormalities in heterozygous animals (Figure 3; Table S2). In contrast, structural analysis of femora from untreated *LMNA* ^G/G^ mice revealed pronounced deficits in trabecular bone. Trabecular bone volume fraction (Tb BV/TV) was reduced by 32% in females (*p* < 0.04) and 55% in males (*p* < 0.01) relative to wild‐type littermates. These reductions were primarily driven by significant decreases in trabecular thickness, which was reduced by 14% in females (*p* < 0.001) and 29% in males (*p* < 0.001). Consistent with these structural changes, trabecular bone mineral density (Tb BMD) was also significantly lower in *LMNA* ^G/G^ mice at six months of age, decreasing by 21% in females (*p* < 0.01) and 42% in males (*p* < 0.001) compared with wild‐type controls. Although cortical tissue mineral density (Ct BMD) was equivalent to wild‐type mice, several cortical geometric parameters were significantly altered in *LMNA* ^G/G^ mice. Medullary area (Ma.Ar) was reduced by 17% in females (*p* < 0.001) and 26% in males (*p* < 0.001) compared to *Lmna* ^+/+^. Similarly, cortical thickness (Ct.Th) was decreased 13% in females (*p* < 0.001) and 9% in males (*p* < 0.01) relative to wild‐type mice. These reductions in cortical geometry resulted in a 38%–46% decrease in the calculated moment of inertia (*p* < 0.001), reflecting the overall smaller femoral structural properties in *LMNA* ^G/G^ mice.

Comparison of P3‐ and P14‐treated mice with untreated *LMNA* ^G/G^ mice showed a trend toward increased trabecular bone volume fraction (Tb BV/TV), with increases of 19% and 24%, respectively. However, these differences did not reach statistical significance due to substantial inter‐mouse variability. Trabecular bone mineral density (Tb BMD) also remained unchanged in both P3‐ and P14‐treated mice relative to untreated *LMNA* ^G/G^ controls. Despite this, trabecular thickness (Tb Th) increased significantly following ABE treatment; Tb Th increased by 16%–24% (*p* < 0.001) in P3‐treated mice and by 11%–19% (*p* < 0.001) in P14‐treated mice. Within the cortical bone compartment, medullary area (Ma.Ar) was further reduced by 11%–14% (*p* < 0.05) in P14‐treated mice, whereas no change was observed in P3‐treated mice compared with untreated *LMNA* ^G/G^ mice. Cortical area (Ct.Ar) increased by 4%–5% (not significant, NS) in P3‐treated mice and by 7%–8% (*p* < 0.05) in P14‐treated mice. These changes were accompanied by increases in cortical thickness (Ct.Th) of 5% (*p* < 0.05) and 9% (*p* < 0.01) in P3‐ and P14‐treated mice, respectively. Although cortical thickness increased in P14‐treated *LMNA* ^G/G^ mice and resulted in greater cortical tissue area, much of this change was associated with a reduction in medullary area, suggesting that additional bone tissue deposition occurred primarily along the endosteal surfaces of the femur in ABE‐treated mice. Nevertheless, these structural changes were insufficient to improve the moment of inertia in either treatment group.

Results of mechanical testing of mouse femora were consistent with the structural analyses that showed normal physical parameters in *LMNA* ^G/+^, but smaller, thinner long bones in *LMNA* ^G/G^ mice (Figure 3, Table S3). Untreated *LMNA* ^G/G^ femora exhibited inferior elastic and plastic properties when compared to wild‐type mouse femora; yield load was decreased by 14% in females (NS) and 34% in males (*p* < 0.01), while stiffness was reduced by 29% in females (*p* < 0.01) and 37% in males (*p* < 0.01). Maximum load was also significantly lower, decreasing by 24% in females (*p* < 0.001) and 30% in males (*p* < 0.001), and fracture load declined by 18% in females (NS) and 28% in males (*p* < 0.05).

ABE treatment partially improved several mechanical parameters. Yield load increased relative to untreated *LMNA* ^G/G^ mice, rising by 12% in females (NS) and 53% in males (*p* < 0.001) in P3‐treated mice, and by 18% in females (NS) and 54% in males (*p* < 0.01) in P14‐treated mice, restoring values to levels comparable with wild‐type mice. Stiffness also increased with treatment, although improvements were modest: 14% in females and 18% in males in P3‐treated mice (NS), and 17% in females (NS) and 19% in males (*p* < 0.05) in P14‐treated mice. Maximum load increased by 9% in females (NS) and 27% in males (*p* < 0.01) in P3‐treated mice, and by 14% in females (NS) and 30% in males (*p* < 0.01) in P14‐treated mice; however, these values remained significantly lower than those of wild‐type femora. Fracture load showed no significant improvement in either treatment group compared with untreated *LMNA* ^G/G^ mice. These changes in physical parameters demonstrate that the *LMNA* ^G/G^ bone phenotype is more severe, and the response to treatment is greater, in males compared to females. Furthermore, these findings suggest that both P3 and P14 ABE treatments partially improve bone mechanical properties, with P14 treatment showing a modestly stronger effect in some parameters, but neither treatment fully restores bone strength to wild‐type levels.

Since the alterations in bone structural parameters occurred primarily on the metaphyseal trabecular and cortical endosteal surfaces, we sought to determine whether these sites correlated with the in vivo location of corrected cells. Immunofluorescent staining of longitudinal sections from P14‐treated mouse femora was performed using antibodies that detect both mouse and human A‐type lamins (4c11) or were specific to human progerin (YZ288). Due to the high level of autofluorescence from heme groups within the marrow compartment, we were unable to visualize any specific cells residing on endosteal cortical or trabecular surfaces that appeared to be negative for progerin. Within the metaphyseal region of femora, however, we identified clusters of osteocytes embedded within the trabeculae that lacked progerin while staining positive for lamins A/C (Figure 2B). Such progerin‐negative but lamin A/C‐positive cells were less abundant in cortical bone and tended to be close to or occasionally residing on periosteal surfaces (Figure 2C). In accordance with the persistent dystrophic growth plate features, no corrected chondrocytes could be identified by immunostaining of growth plates and articular cartilage of ABE‐treated mice (data not shown). These data are consistent with improvements to bone structural and mechanical properties in ABE‐treated *LMNA* ^G/G^ mice that are driven by pockets of corrected osteoblasts and/or osteocytes within cortical and trabecular tissues.

To further characterize mechanisms underlying bone dysplasia in HGPS mice, gene expression in tibial bone tissue from wild‐type, untreated, and P14 ABE‐treated *LMNA* ^G/G^ littermates was analyzed. Differential expression analysis (DGEA) was performed on bulk RNA sequencing data and identified 10,151 genes (FDR < 0.05) with transcript levels that were significantly altered in six month‐old *LMNA* ^G/G^ mice compared to wild‐type (Supporting Data File S1). Using Gene Ontology (GO) terms to identify biological processes (BP) enriched with differentially expressed genes, gene set enrichment analysis (GSEA) revealed 902 highly redundant biological processes that were significantly altered in *LMNA* ^G/G^ bone tissue versus wild‐type mice (FDR < 0.05) (Supporting Data File S2). Gene sets associated with cellular respiration, nucleotide triphosphate metabolism, intracellular calcium transport, skeletal and cardiac muscle development and function, cellular responses to growth factors, and broader skeletal system development were enriched and overrepresented in the *LMNA* ^G/G^ transcriptome compared to wild‐type mice. Significantly increased enrichment scores were identified for processes involved in skeletal system development including ossification, odontogenesis, biomineral tissue development, bone and dentin mineralization, amelogenesis, and cranioskeletal system development, likely due to dysregulation of BMP and WNT signaling, and osteoblast, osteocyte and odontoblast differentiation. GO processes involved in osteoclast proliferation and differentiation were also upregulated. In contrast, gene expression programs involved in immune responses, DNA replication and checkpoint signaling, cartilage development, and epidermal growth factor signaling were significantly under‐represented in *LMNA* ^G/G^ bone (Supporting Data File S2).

**FIGURE 4:**
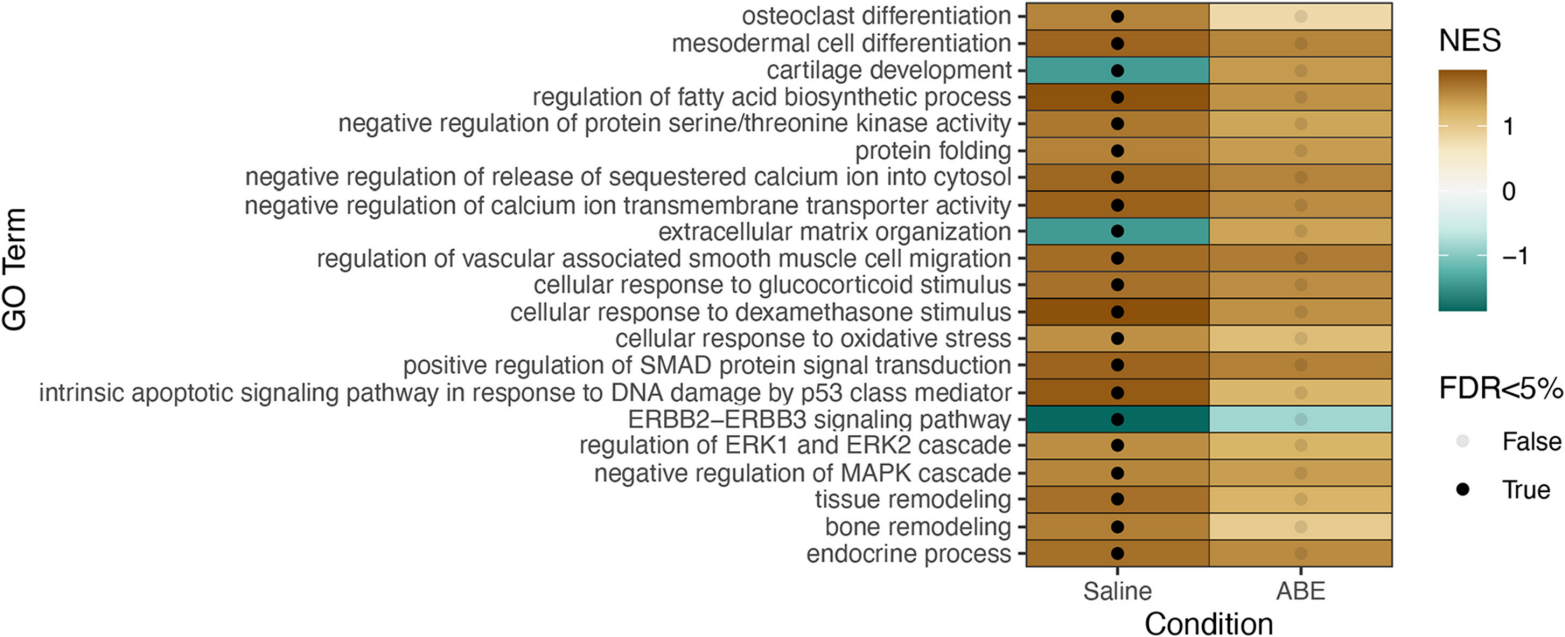
Representative pathways normalized by *LMNA* gene correction in murine bone tissue. Heat map of GSEA results showing rescued bone‐relevant Gene Ontology biological processes from bulk six‐month tibial bone RNA sequencing data. Full analysis reporting normalized enrichment scores (NES) and statistical significance versus wild‐type mice is reported in Supporting Data File S2. *N* = 12 (six males, six females) for wild‐type (WT), P14 saline‐treated *LMNA* ^G/G^ (Saline) and P14 ABE‐treated *LMNA* ^G/G^ (ABE) mice at six months of age.

We next filtered GSEA results for pathways that were significantly altered in *LMNA* ^G/G^ mouse bone but normalized toward wild‐type levels in ABE‐treated mice and identified 103 overlapping GO biological processes that were identified as candidate pathways involved in the partial bone phenotypic rescue of *LMNA* ^G/G^ mice following ABE treatment (Supporting Data File S2). Our analyses indicated normalization of pathways relevant to skeletal system development including osteoclast differentiation, cartilage development, SMAD signaling, MAPK–ERK signaling, extracellular matrix organization, and bone and tissue remodeling (Figure 4). Additional normalized pathways involved p53 signaling, apoptotic processes, steroid responses, post‐translational modifications associated with kinase activity, intracellular calcium flux, and ERBB signaling.

**FIGURE 5:**
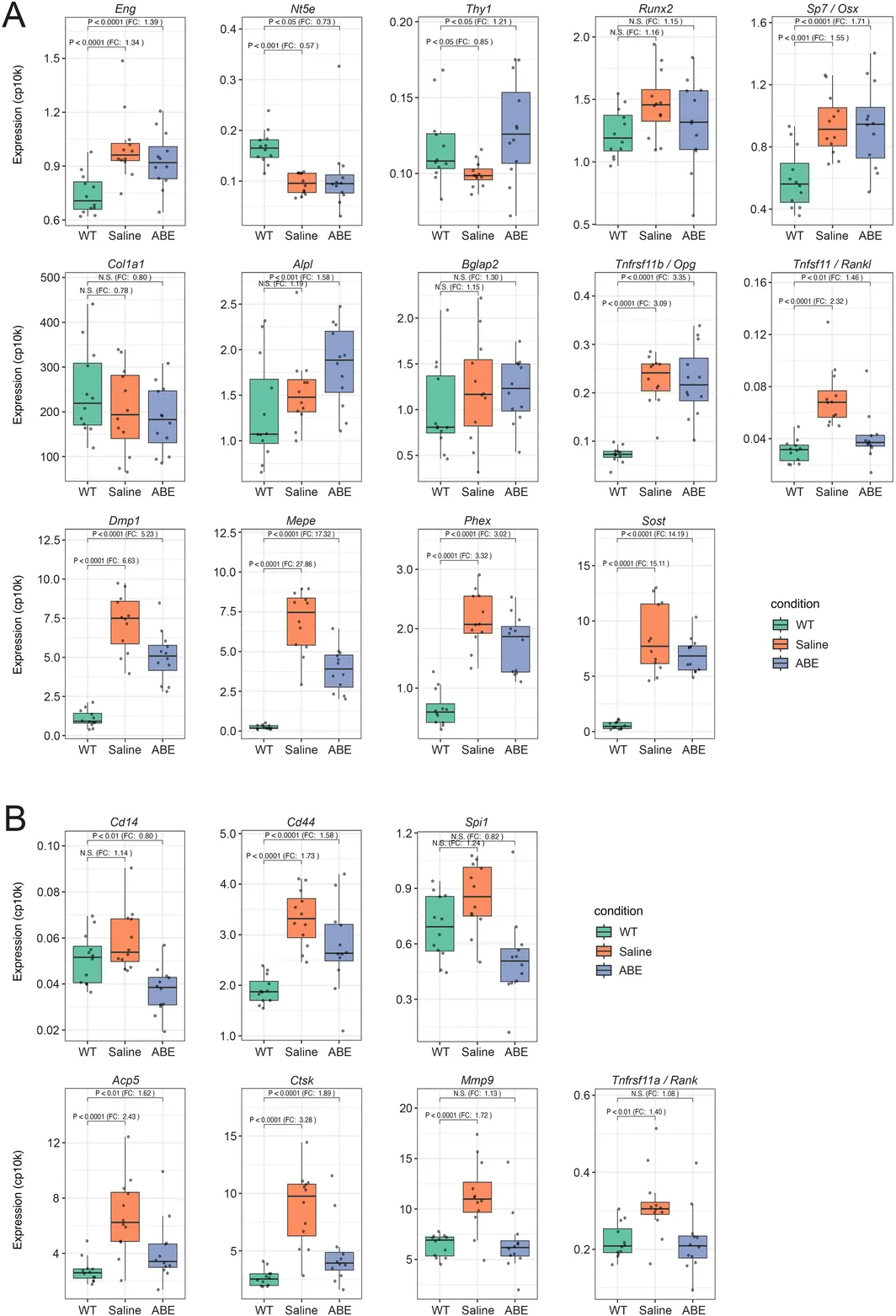
Differential expression analysis shows normalization of markers of bone cell populations in ABE‐treated *LMNA* ^G/G^ mice. (A) Quantitation of transcripts from murine bone tissue shows dysregulated expression of some early (top), middle (center) and late markers (bottom) markers of the mesenchyme‐derived osteogenic lineage required for differentiation, cellular crosstalk and matrix mineralization. (B) Markers of hematopoietic lineage osteoclasts. Increased expression of mononuclear precursor‐associated (top) and mature, bone‐resorbing osteoclast genes (bottom) in saline‐treated mice are reduced to wild‐type (WT) levels in ABE‐treated (ABE) mice. *N* = 12 (six males, six females) for wild‐type (WT), P14 saline‐treated *LMNA* ^G/G^ (Saline) and P14 ABE‐treated *LMNA* ^G/G^ (ABE) mice at six months of age. FC, fold‐change. *p* values versus wild‐type are shown within each graph. N.S., not significant.

Given the large number of genes whose expression appeared to be altered in *LMNA* ^G/G^ mice, we focused on transcripts known to be critical for bone development and homeostasis (Figure 5, Supporting Data File S1). Osteoblast‐specific genes (*Runx2*, *Sp7/Osx*) tended to be upregulated in *LMNA* ^G/G^ bone tissue but were not reduced in ABE‐treated mice (Figure 5A). Notably, we found that both *Rankl* and *Opg* expression were significantly increased in *LMNA* ^G/G^ mice, and their expression ratio decreased by 42% in ABE‐treated mouse bone tissue, reflecting normalization of *Rankl* transcripts to wild‐type levels (Figure 5A). This observed down‐regulation of *Rankl* would be expected to affect the activation of hematopoietic lineage osteoclast precursors in treated mice. Indeed, in saline‐treated *LMNA* ^G/G^ mice, expression of mononuclear osteoclast precursor markers (*Cd14*, *Spi1*, and *Cd44*) trended toward higher expression than wild‐type mice (Figure 5B). We found, however, that expression of markers indicative of mature, multinucleated osteoclasts (*Tracp5/Acp5*, *Ctsk*, *Mmp9*, and *Tnfrsf11a/Rank*) was significantly increased in *LMNA* ^G/G^ mice, and the reduced *Rankl/Opg* transcript ratio in ABE‐treated mice was reflected in the downregulation of these genes (Figure 5B). Markers of osteocytes (*Dmp1*, *Mepe*, *Phex*, and *Sost*), an additional source of soluble RANKL in vivo (Nakashima et al. 2011), were uniformly upregulated in *LMNA* ^G/G^ mouse bone but were expressed at lower levels in *LMNA* ^G/G^ mice treated with ABE (Figure 5A).

**FIGURE 6:**
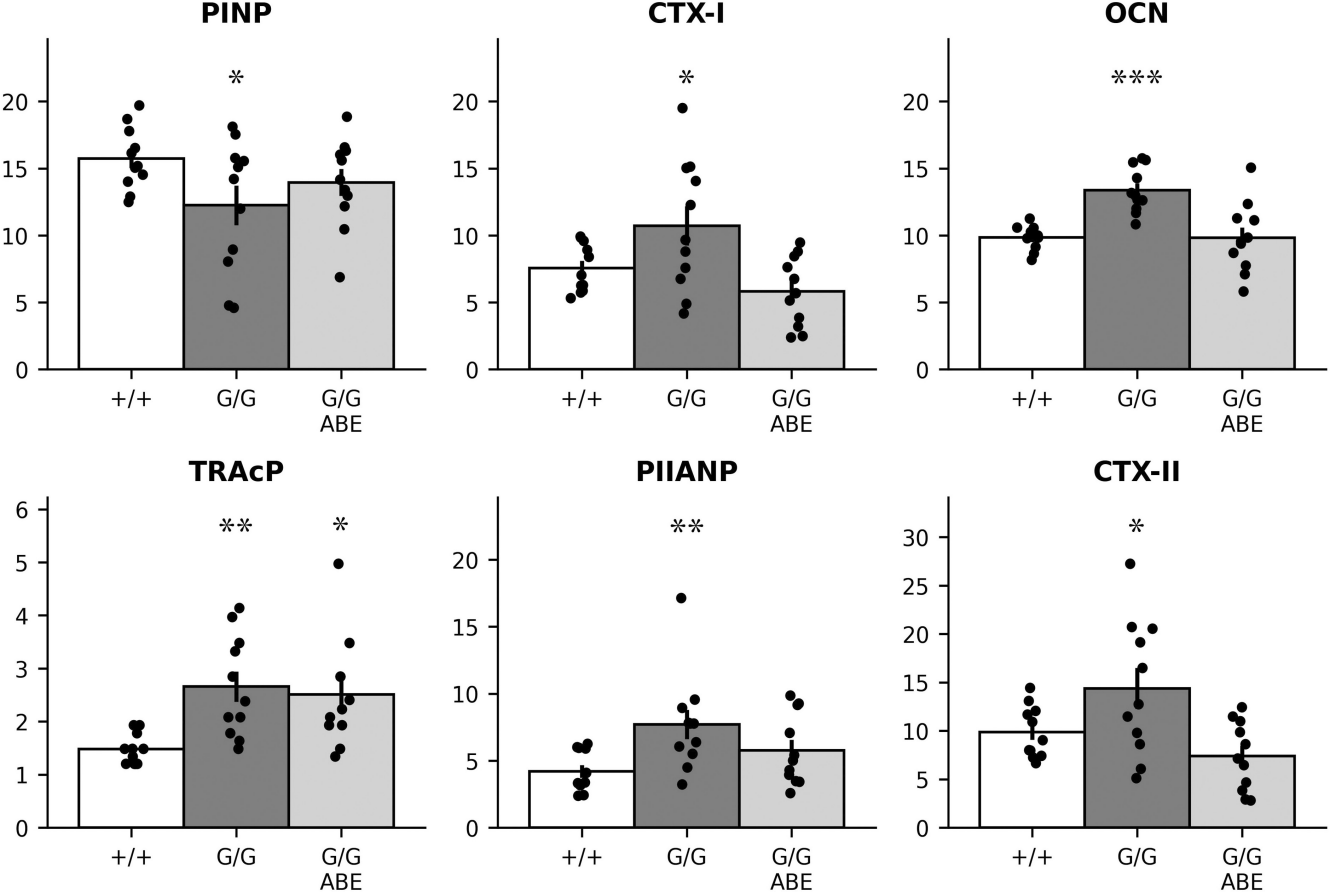
Circulating markers of bone and cartilage formation and degradation are normalized by ABE treatment in *LMNA* ^G/G^ mice. Reduced plasma levels of procollagen I N‐propeptide (PINP) and increased crosslinked collagen I C‐telopeptide (CTX‐I), osteocalcin (OCN), and tartrate‐resistant acid phosphatase (TRAcP) are consistent with lower bone formation and higher resorption in *LMNA* ^G/G^ mice but are restored to wild‐type (+/+) levels in treated (ABE) mice. Elevated plasma procollagen IIA N‐propeptide (PIIANP) and crosslinked Type II collagen C‐telopeptide indicate active articular cartilage remodeling in *LMNA* ^G/G^. *N* = 12 (six males, six females) for wild‐type (+/+), P14 saline‐treated *LMNA* ^G/G^ (G/G), and P14 ABE‐treated *LMNA* ^G/G^ (G/G ABE) mice. *p < 0.05; **p < 0.01; ***p < 0.001 versus *Lmna* ^+/+^ littermates.

Noting the transcriptional differences between treated and untreated *LMNA* ^G/G^ bone, we considered whether these changes reflected shifts in bone cell population composition or reprogramming of gene expression following *LMNA* correction, either of which could alter mesenchymal and hematopoietic lineage cell differentiation and function during tissue remodeling in ABE‐treated *LMNA* ^G/G^ mice. Specific markers of bone and cartilage metabolism were analyzed in plasma collected from mice included in this study. Levels of Type I (PINP) and Type II procollagen (PIIANP) N‐propeptides indicative of osteoblast and chondrocyte activity, respectively, were significantly altered in saline‐treated *LMNA* ^G/G^ mice and were restored to wild‐type levels in ABE‐treated mice (Figure 6). Elevated markers of bone resorption (CTX‐I) and cartilage degradation (CTX‐II) in *LMNA* ^G/G^ were reduced in the ABE treatment group, while tartrate‐resistant acid phosphatase (TRAcP) levels remained increased following treatment. Although osteocalcin (OCN) is produced primarily by osteoblasts in vivo, it is also released from bone matrix upon resorption; we found that significantly increased levels of this protein were reduced to wild‐type levels in the ABE treatment group. Accordingly, the plasma profile of bone metabolic markers in *LMNA* ^G/G^ mice is concordant with an imbalance between bone formation and resorption, which is normalized by ABE‐mediated transgene correction in treated mice. Taken as a whole, these data are consistent with a rebalancing of cell populations, along with transcriptional changes, in response to ABE treatment.

We also sought to determine the mechanism underlying the increase in the extent of corrected transgenes in bone tissue of six month‐old, compared to six week‐old, treated mice (Koblan et al. 2021). As observed in the DGE analysis, no consistent difference in the expression of markers of mesenchymal stem cells (*Thy1*, *Nt5e*, *Eng*) was found between P14 saline‐treated and ABE‐treated *LMNA* ^G/G^ mice (Figure 5A). However, residual expression of both the N‐ and C‐terminus of the dual‐vector base editing system in bone tissue was confirmed in six month‐old P14 treated mice, which corresponded to a 56% ± 19% increase in *LMNA* (*p* < 0.02) and a 47% ± 5% decrease in progerin (*p* < 0.001) transcripts compared to untreated mice (Figure S5). Although we cannot rule out expansion or in vivo selection of the stem cell population following transgene correction, our data are consistent with an interpretation that continued expression of the base editing system nearly six months post‐treatment may have contributed to the increased fraction of corrected transgenes as the mice aged.

## 3 Discussion

In this study, we used the double‐copy transgenic mouse (*LMNA* ^G/G^) to characterize skeletal pathology in HGPS, examine its relevance to aging‐related bone loss, and evaluate the effects of base‐editing correction in vitro and in vivo. Analyses were conducted at six months of age, when systemic disease is well established but precedes mortality at approximately eight months (Cabral et al. 2021). At this stage, untreated mice displayed pronounced trabecular deficits, including reduced bone volume (BV/TV) and mineral density (BMD), along with an increased structural model index (SMI), indicative of a more rod‐like, age‐associated architecture. Although cortical BMD remained unchanged, decreased cortical thickness and marrow area reflected thinner long bones and reduced mechanical strength, extending prior reports of progressive skeletal deterioration in this model (Cabral et al. 2021).

The findings reported in this study suggest a model with parallels to aging‐related osteoporosis, in which reduced bone formation combined with increased resorption are among the factors contributing to the *LMNA* ^G/G^ skeletal phenotype. Histologic analysis revealed increased empty osteocyte lacunae, a feature shared with other HGPS models and normal aging, that is potentially driven by progerin‐induced senescence, accelerated differentiation, and premature apoptosis of mesenchymal‐derived bone cells. Impaired osteocyte mechanosensing may further compromise cell survival (Barettino et al. 2024; Blouin et al. 2024, 2023; Galli et al. 2010; Osmanagic‐Myers et al. 2015; Schmidt et al. 2012; Tiede‐Lewis et al. 2017). Furthermore, the concurrent upregulation of osteoclast‐specific genes and increased circulating resorption markers (CTX‐I, TRAcP) would be consistent with heightened osteoclastic activity, which remains to be experimentally validated.

In multiple HGPS mouse models, including *LMNA* ^G/G^ transgenic mice, the skeletal phenotype is characterized by loss of osteoblasts and osteocytes, impaired matrix deposition, compromised mechanical integrity, and disrupted endochondral ossification. In the *Lmna* ^G609G/G609G^ knock‐in model, bone dysplasia results from defective osteoblast differentiation, osteocyte loss, and suppression of osteoclastogenesis, leading to reduced bone deposition with relatively normal resorption and diminished femoral mechanical properties associated with altered matrix composition (Blouin et al. 2024; Cabral et al. 2023; Osorio et al. 2011; Zaghini et al. 2020). Growth plate disorganization and increased apoptosis of proliferative and hypertrophic chondrocytes further indicate impaired endochondral bone development (Blouin et al. 2024). Similarly, inducible osteoblast‐specific *LMNA* c.1824C>T, *Lmna* ^−/−^, as well as *Lmna* ^HG/+^ mice that express progerin exclusively from one allele, exhibit reduced bone mass, decreased bone cell numbers, low bone turnover, and compromised mechanical strength (Li et al. 2011; Schmidt et al. 2012; Yang et al. 2006). Collectively, these models demonstrate that impaired osteoblast function and reduced bone formation are central features of HGPS‐associated skeletal disease, but distinguish them from the distinct remodeling phenotype observed in the humanized *LMNA* ^G/G^ transgenic model.

Treatment of *LMNA* ^G/G^ mice via P14 AAV9‐mediated delivery of ABE resulted in approximately 11%–22% correction of transgene copies in bone at six weeks and six months of age. This level of correction presumably corresponds to the fraction of the bone cell population that is edited, assuming that both transgene copies within a double‐copy cell are corrected upon internalization and expression of the ABE. However, in the event that only one transgene copy per cell is corrected in vivo, the resulting 22%–44% of heterozygous cells would still be expected to exhibit improved function compared to uncorrected cells based on the absence of pathology outside of the vascular system in single‐copy transgenic mice (*LMNA* ^G/+^) (Varga et al. 2006). Regardless of the correction mechanism, the relatively modest level of transgene correction in bone cell populations still led to alterations in some structural and physical parameters of ABE‐treated *LMNA* ^G/G^ mice. These changes included increased trabecular (Tb Th) and cortical thickness (Ct Th), resulting in improved yield load and stiffness, though moment of inertia, fracture load, and overall femoral size were not significantly improved, indicating that architectural gains were insufficient to translate into full mechanical recovery.

Although it is difficult to extrapolate gene‐editing efficiency in tibiae to histologic observations in femora, the sites of edited bone cells mostly corresponded to regions of the femora showing improvement in structural parameters. Furthermore, it is possible that some cells staining positive for progerin had been corrected by the ABE, but the longer half‐life of progerin compared to normal lamin A, and the non‐proliferative nature of osteocytes, prevented their detection (Puttaraju et al. 2021). We therefore speculate that ABE‐mediated partial correction contributes to the improvements that were detected at least in part through effects on osteocyte function, potentially rebalancing paracrine regulation of bone turnover. Improved osteocyte viability and signaling could plausibly reduce the *Rankl*/*Opg* ratio, attenuate RANK‐RANKL‐dependent osteoclastogenesis, and normalize resorptive activity, though the precise relationship between these factors remains to be established. Additionally, reduced osteoclast activity may reflect, in part, editing of marrow‐derived progenitors, potentially limiting their differentiation or function. These interpretations are supported by, though not fully explained by, normalization of osteocyte‐ and osteoclast‐related gene expression, resorption‐associated GO pathways, and circulating bone and cartilage degradation markers.

Genetic mosaicism provides important guidance for gene therapy strategies. Studies of somatic carriers with bone dysplasias suggest that partial correction of dominant‐negative mutations can substantially attenuate disease severity, as a significant fraction of mutant bone cells may still be compatible with relatively normal skeletal function compared to the severe phenotypes seen in heterozygous offspring (Cabral and Marini 2004; Desir et al. 2012). Gene therapy for skeletal disorders, however, remains challenging due to the relative avascularity of bone and cartilage, which limits efficient transduction of chondrocytes and mineral‐embedded osteocytes. Although AAV9 can target osteoblasts, osteoclasts, and osteocytes in cortical and trabecular bone, it shows limited transduction of ligament, articular cartilage, and growth plate tissues (Lee et al. 2019; Yang et al. 2019, 2020). Nonetheless, AAV‐based gene addition and silencing strategies have demonstrated efficacy in preclinical skeletal disease models, including reversal of bone and cartilage pathology in lysosomal storage disorders and suppression of heterotopic ossification in fibrodysplasia ossificans progressiva (Bertolin et al. 2021; Ko et al. 2016; Yang et al. 2023). To our knowledge, this study is the first to demonstrate successful AAV‐mediated in vivo delivery of a DNA base editor to bone, and that incomplete ABE‐mediated correction may still be sufficient for therapeutic benefit.

Future inclusion of the bone‐targeting peptide motif (Asp‐Ser‐Ser)_6_ within the capsid protein of AAV9 or other gene delivery vectors might significantly enhance bone tropism. Such use of bone‐targeting motifs has already proven effective in pre‐clinical and clinical trials investigating enzyme replacement therapy in mice and children with hypophosphatasia (Millan et al. 2008; Whyte et al. 2016). Additional factors affecting the efficacy of AAV9‐mediated transduction of bone tissue in the current study may include the method of delivery (i.e., intravenous vs. retro‐orbital), variability among specific viral preparations, and the age at which mice were treated. For example, P14 injections generally achieved higher base‐editing efficiencies than P3 injections, possibly due to the ten‐fold higher AAV dose that could be injected into larger mice, as well as increased expression of AAV9 receptors in older mice (Bostick et al. 2007; Levy et al. 2020; Wang et al. 2012). It is also possible that the higher rate of editing observed in P3‐ and P14‐treated mice, compared to lower editing rates in mice treated at one and four months of age, was due to the increased time for additional editing to occur, somatic selection of edited cells, or exhaustion of osteoblast progenitors in vivo.

We further note that male *LMNA* ^G/G^ mice showed a consistently stronger response to ABE treatment than females across mechanical parameters, a disparity that may reflect the more severe baseline bone phenotype in males that would render equivalent editing rates more detectably impactful, as well as sex‐based biological differences in therapeutic response. It is possible that stronger humoral and cellular immune responses in females may reduce functional AAV delivery to target bone cells, while hormonal influences on osteoblast activity and transgene expression could further limit correction efficiency (Khosla and Monroe 2018; Warrington et al. 2025). Collectively, these factors may converge to produce the sex‐dependent pattern observed here, suggesting that strategies to enhance AAV bone tropism warrant consideration for improving therapeutic outcomes in female subjects.

In summary, although bone is not the tissue of primary concern in HGPS, our initial in vivo base‐editing approach achieved up to 22% gene correction in *LMNA* ^G/G^ mice, a level sufficient to partially rescue structural and functional skeletal defects. While more extensive editing may be required for disorders with more severe bone pathology, these findings establish proof‐of‐concept that a locus‐specific base editor can be successfully delivered to mineralized bone and suggest that this platform could be adapted for other monogenic bone disorders. As advances in treating the vascular complications of HGPS extend survival, preservation of skeletal integrity will become increasingly important. Notably, if bone represents one of the more challenging tissues to transduce, the functional improvements observed here imply that even greater therapeutic benefit may be achievable in tissues with higher editing efficiency. Moreover, improving bone health has systemic implications, given the reciprocal interactions between bone, vascular biology, and energy metabolism in progeria and other genetic disorders.

## 4 Experimental Procedures

Experimental procedures and materials used in this study can be found in the Supplemental files accompanying this article.

## Author Contributions

W.A.C., C.M.G., S.M.W., N.N., D.R.L., A.N., M.R.E., and F.S.C. designed the experiments, which were conducted by W.A.C., D.Y., I.B., S.L., L.R.J., U.L.T., L.W.K., X.D.G., and M.A. Data were analyzed by W.A.C., C.M.G., D.Y., I.B., S.L., L.R.J., T.Y., L.W.K., X.D.G., M.A., and N.N. The manuscript was written by W.A.C. and F.S.C., with significant input from M.R.E., N.N., L.G.B., D.R.L., and A.N.

## Funding

This work was supported by the National Human Genome Research Institute (Grant NHGRI HG200305).

## Conflicts of Interest

D.R.L. is a co‐founder and equity holder of Beam Therapeutics, Prime Medicine, Pairwise Plants, and nChromaBio, companies that use or deliver gene editing or epigenome‐modulating agents. L.G.B. receives research support from Merck Inc. and royalties from Wolters Kluwer.

## Supporting information


**Table S1:** Transgene correction at 6 months following ABE treatment at 1 and 4 months.
**Table S2:** *Lmna* ^+/+^, *LMNA* ^G608G/+^ and *LMNA* ^G608G/G608G^ femoral bone structural parameters.
**Table S3:** *Lmna* ^+/+^, *LMNA* ^G608G/+^ and *LMNA* ^G608G/G608G^ femoral bone physical parameters.
**Figure S1:** Confocal immunofluorescence microscopy shows no detectable progerin in ABE‐corrected *LMNA* ^G/G^ osteoblasts in vitro. Cells were imaged following treatment of wild‐type (+/+) and *LMNA* ^G/G^ (G/G) osteoblasts with saline, or *LMNA* ^G/G^ osteoblasts treated with ABE (G/G ABE). Staining was performed with DAPI to visualize nuclei, phalloidin to visualize cytoplasmic area, and progerin‐specific antibody. Nuclear blebbing is evident in untreated *LMNA* ^G/G^, but not in wild‐type or ABE‐treated cells. Scale bar = 20 μm.
**Figure S2:** Quantitation of gene correction in *LMNA* ^G/G^ osteoblasts in vitro. (A) Left, Validation of droplet digital PCR SNP assay for *LMNA* c.1824C>T allele quantitation. A standard curve was generated using sequential dilutions of normal human and double copy murine transgenic fibroblast genomic DNA. Right, quantitation of C and T alleles in osteoblast cultures treated at a range of increasing MOI. NT, no treatment. (B) Quantitative RT‐PCR results of transgene‐derived *LMNA* transcript copy number (left) and specific transcripts expressed as a fraction of total transgene‐derived *LMNA* transcripts (right). Transgene correction levels are denoted in red. (C) Quantitation of transgene‐derived protein products based on Western analysis results. Levels of LMNA (A), progerin (P) and LMNC (C) are expressed as a fraction of total transgene‐derived A‐type lamins (left) or as a ratio relative to LMNC (right). *p < 0.05; **p < 0.01; ***p < 0.001.
**Figure S3:** Femoral growth plate histology of female mice. Growth plates were stained with Safranin‐O/Fast Green to identify cartilage proteoglycans (red) and mineralized bone tissue (green). Epiphysis and metaphysis are located to the left and right, respectively, of the growth plate. The cartilage to bone transitional zone is observed as red‐stained (Safranin‐O positive) protrusions intruding into the ossified (green) tissue. Scale bar = 100 μm.
**Figure S4:** Femoral growth plate histology of male mice. Growth plates were stained with Safranin‐O/Fast Green to identify cartilage proteoglycans (red) and mineralized bone tissue (green). More intense Safranin‐O staining is observed in the hypertrophic region of growth plates of untreated male *LMNA* ^G/G^ mice. Scale bar = 100 μm.
**Figure S5:** Adenine Base Editor expression remains active in bone at six months post‐treatment. (A) Gene expression analysis of RNA sequencing reads confirms continued expression of the dual vector ABE7.10 base editor system in vivo at six months of age in P14‐treated murine bone tissue. The C‐terminal portion of the dual vector ABE system expresses 2.85 ± 0.31 ‐fold more than the N‐terminal fragment at this age. (B) Quantitation of transcripts from bulk RNA sequencing data showing increased *LMNA* (*p* < 0.02) and decreased progerin (*p* < 0.001) expression in bone tissue of P14‐treated mice at six months of age. Saline, *LMNA* ^G/G^ mice injected at P14 with saline only; ABE, *LMNA* ^G/G^ mice treated at P14 with ABE.

## Data Availability

RNA sequence reads and gene quantification data are available at GEO with accession number GSE334271. All other data that support the findings of this study are available from the authors upon request.
