# Supplementary material for: In Vivo Base Editing Partially Rescues Bone Dysplasia in a Mouse Model of Hutchinson‐Gilford Progeria Syndrome
Source: Aging Cell. 2026 Sep 3;25(9):e70700. doi: 10.1111/acel.70700 (PMC13539538; doi:10.1111/acel.70700)
Supplement: Supplementary file 1 [file ACEL-25-e70700-s001.docx]

***In vivo* base editing partially rescues bone dysplasia in a mouse model of Hutchinson-Gilford Progeria Syndrome.**

Wayne A. Cabral^1#^, Caleb M. Grenko^1^, Diana Yeritsyan^2^, Indeevar Beeram^2^, Shubham Laiwala^2^, Lukas R. Johnson^1^, Urraca L. Tavarez^1^, Tingfen Yan^1^, Stephen M. Wincovitch^3^, Luke W. Koblan^4,5,6^, Xin D. Gao^4,5,6,7^, Meirui An^4,5,6^, Narisu Narisu^1^, Leslie G. Biesecker^1^, David R. Liu^4,5,6^, Ara Nazarian^2^, Francis S. Collins^1,8^, and Michael R. Erdos^1^

^1^ Molecular Genetics Section, Center for Precision Health Research, National Human Genome Research Institute, NIH, Bethesda, MD, USA

^2^ Musculoskeletal Translational Innovation Initiative, Carl J. Shapiro Department of Orthopedic Surgery, Beth Israel Deaconess Medical Center and Harvard Medical School, Boston, MA, USA

^3^ Advanced Imaging and Analysis Core Facility, National Human Genome Research Institute, NIH, Bethesda, MD, USA

^4^ Merkin Institute of Transformative Technologies in Healthcare, Broad Institute of Harvard and MIT, Cambridge, MA, USA

^5^ Department of Chemistry and Chemical Biology, Harvard University, Cambridge, MA, USA

^6^ Howard Hughes Medical Institute, Harvard University, Cambridge, MA, USA

^7^ Current address: Department of Ophthalmology, University of Pittsburgh School of Medicine, Pittsburgh, PA, USA

^8^ Current address: The Progeria Research Foundation, Peabody, MA, USA

**Supporting Information**

Experimental Procedures

Table S1. Transgene correction at 6 months following ABE treatment at 1 and 4 months.

Table S2. *Lmna*^+/+^, *LMNA*^G608G/+^ and *LMNA*^G608G/G608G^ femoral bone structural parameters.

Table S3. *Lmna*^+/+^, *LMNA*^G608G/+^ and *LMNA*^G608G/G608G^ femoral bone physical parameters.

Figure S1. Confocal immunofluorescence microscopy shows no detectable progerin in ABE-corrected *LMNA*^G/G^ osteoblasts *in vitro*.

Figure S2. Quantitation of gene correction in *LMNA*^G/G^ osteoblasts *in vitro*.

Figure S3. Femoral growth plate histology of female mice.

Figure S4. Femoral growth plate histology of male mice.

Figure S5. Adenine Base Editor expression remains active in bone at six months post-treatment.

**Experimental Procedures**

**Mouse Model.** Our transgenic mouse model of HGPS was developed by retrofitting a human bacterial artificial chromosome (BAC) harboring the *LMNA* gene containing the classic G608G mutation (c.1824C>T) and a second functional gene, *RAB25*, as previously described (Cabral *et al.*, 2021; Varga *et al.*, 2006). Single-copy mice, designated *LMNA*^G/+^, were bred within the C57BL/6J line for 20 generations prior to generation of double-copy mice, designated as *LMNA*^G/G^, for experimental use. Mice used in this study were treated at specified ages by retro-orbital injection of split AAV constructs at 3 days (P3; n=12; six males, six females; 10^11^ vg), 14 days (P14; n=12; six males, six females, 10^12^ vg), 1 month (1M; n=6; three males, three females, 1.48 × 10^14^ vg) and 4 months of age (1M; n=6; three males, three females, 1.48 × 10^14^ vg). Animal care and experiments were performed in accordance with a protocol approved by the NHGRI Animal Care and Use Committee.

**AAV9 and Lentiviral Production.** AAVs were generated and quantified as previously described (Koblan *et al.*, 2021; Levy *et al.*, 2020). Lentivirus expressing ABE7.10max was generated by insertion of the ABEmax-VRQR gene and LMNA c.1824C>T sgRNA into the lentiCRISPRv2 backbone (Addgene) (Koblan *et al.*, 2021). HEK293T/17 (ATCC) cells, approximately 70% confluent, were transfected using FuGENE HD according to the manufacturer’s protocol (Promega). Transfection mix included 9 mg of transfer vector (pLentiCRISPr-ABE7.10maxG608G), 9 mg of psPAX2 (encoding the viral packaging proteins) and 6 mg of pVSV-G (encoding the VSV-G envelope protein). The transfection mix was then supplemented with 70 ml of room-temperature FuGENE and brought to a final volume of 1,500 ml per flask with Opti-MEM (ThermoFisher Scientific). Two days after transfection, media were collected, centrifuged at 3,000 × g for 15 minutes, and passed through a 0.45 μm PVDF filter to remove non-viral debris. Supernatant containing lentivirus was added directly to cell cultures.

Lentiviral titration was performed using the BioRad QX200 Droplet Digital PCR system and a custom assay targeting copies of the Rev response element (RRE). In brief, RNA was isolated from 1 ml of media using TRIzol LS reagent (ThermoFisher Scientific) and reverse-transcribed with the iScript cDNA Synthesis Kit (BioRad). Following droplet generation, serial ten-fold dilutions of resuspended viral RNA were amplified with 900 nM forward primer (5’-tgtgccttggaatgctagt-3’), 250 nM probe (5’-FAM-tttggaatcacacgacct-3’) and 900 nM reverse primer (5’-aatttctctgtcccactccatc-3’) under standard conditions (Wang *et al.*, 2018). RRE copy number concentration per ml of initial media was determined after adjusting for dilutions.

**Cell Culture.** Primary osteoblast cultures were generated from newborn mouse calvaria by collagenase release and cultured in αMEM containing 10% fetal bovine serum, 2 mM glutamine, and 1% pen-strep at 37 ^o^C in 8% CO_2_ (Bakker & Klein-Nulend, 2012). Cells undergoing osteogenic differentiation were supplemented with 50 μg/ml ascorbate and 2.5 mM β-glycerophosphate for 21 days, fixed in 4% paraformaldehyde in PBS, followed by staining with 1% alizarin red S in 2% ethanol to reveal mineral. Alizarin Red was solubilized with 0.5 mL of 0.5 N HCl/5% sodium dodecyl sulfate (SDS), and absorbance was measured at 405 nm with a spectrophotometer for quantitation (Gregory *et al.*, 2004).

**SNP Assay.** Genomic DNA was isolated using the Puregene DNA Isolation Kit (Qiagen). The *LMNA* c.1824C>T variant allele fractions were determined by ddPCR using 50 ng of genomic DNA amplified with a custom SNP assay (*LMNA* c.1824C>T, Organism Unspecified Assay, ID #dMDS212771753, BioRad) under standard amplification conditions, and by high-throughput sequencing as described (Koblan *et al.*, 2021). SNP assay validation was performed using a standard curve generated from various mixtures of normal human control and double-copy transgenic murine fibroblast genomic DNA (**Supplemental Figure S2A**).

**Gene Expression Analyses.** Total RNA was extracted from cell cultures or tibial bone tissue after removal of epiphyses and marrow from 12 wild-type (*Lmna*^+/+^, six males, six females), 12 double-copy transgenic mice treated with saline (*LMNA*^G608G/G608G^, six males, six females) and 12 ABE-treated double-copy mice (six males, six females) at six months of age. For bone tissue, epiphyses and marrow were carefully removed prior to RNA extraction, resulting in mostly cortical bone that would be expected to exhibit a gene expression profile representing a cell population enriched for osteocytes (~90-95%), osteoblasts (~4-6%), and osteoclasts (~1-2%), with smaller contributions representing endothelial cells and pericytes in healthy animals (Parfitt, 1977). RNA isolation was performed guanidinium thiocyanate isolation (TRIzol reagent, ThermoFisher Scientific), followed by digestion with DnaseI (ThermoFisher Scientific) for 20 minutes at 37^o^C, then analysis for integrity and concentration on an Agilent Nucleic Acid Bioanalyzer (Agilent Technologies). Quantitation of transgene-derived *LMNA*, *Progerin* and *LMNC*, and endogenous gene-derived transcripts was performed by RNA sequencing and ddPCR as previously described (Cabral *et al.*, 2021).

In brief, sequencing was performed on a sequence by synthesis instrument (Illumina NovaSeq 6000, La Jolla, CA), strand-specific PolyA with 2 × 151 bp cycles. Paired-end RNA-seq reads were aligned to GRCm38 genome assembly using STAR (v2.7.3) with default parameters (Dobin *et al.*, 2013). Expression levels of Gencode M24 genes were quantified using QoRTs (v1.3.6) (Hartley & Mullikin, 2015). On average, we generated 171,159,844 (116,648,352 - 264,3371,606) paired-end reads per library, of which 83.9% were aligned to the genome, indicating high-quality data. Variance stabilizing transformation was performed via the “vst” function of DESeq2 (v1.34) on the raw read count matrix, considering library size, followed by Principal Component Analysis (PCA) using the “PCAplot” of DESeq2 on normalized data, which identified no outlier samples. RNA sequencing reads were used to generate a reference FASTA file of human transcripts (117 bp) unique to humans but absent in mice, and aligned with the “BWA” tool using the “-M” option (v0.7.17-r1194) (Li & Durbin, 2009). Reads with mapping quality score <30 were removed, while those with a perfect match to the human progerin transcripts were retained. We quantified human *LMNA* and *Progerin* expression as the proportion of reads mapped to the transgene to the total number of reads.

For differential expression analysis (DEA), individual experimental conditions (Saline, ABE) were compared versus wild-type (WT) using DESeq2 (version 1.42.0) in R (version 4.3.2). Raw read counts were normalized to library size and quantified per 10,000 reads (cp10k). Genes with an average expression of less than 0.25 cp10k across all samples were excluded. Dispersion was estimated using DESeq2's parametric method with sex as a covariate. P-values were adjusted for multiple testing using the Benjamini-Hochberg procedure in the “stats” R package. Genes with FDR < 0.05 were considered as differentially expressed.

Gene Set Enrichment Analysis (GSEA) was performed using the clusterProfiler R package (version 4.12.2) to identify enriched Gene Ontology (GO) biological process terms among differentially expressed genes in each comparison. For each condition, ranked gene lists based on log2-fold change values were analyzed with the “gseGO” function using ENSEMBL gene IDs (org.Mm.eg.db, version 3.19.1). Empirical p-values were computed with 10,000 permutations using the fgsea algorithm (version 1.30.0) and adjusted by the Benjamini-Hochberg method. GO terms with an FDR < 0.05 were considered enriched. Redundant GO terms were clustered using simplifyEnrichment (version 1.14.0) with the “Rel” similarity measure and “apcluster” algorithm (version 1.4.13). Results were visualized using ggplot2 (version 3.5.1), ggnewscale (version 0.5.0), and ggh4x (0.2.8). ENSEMBL gene IDs were converted to gene symbols using the biomaRt R package (version 2.60.1).

**Biochemical Studies.** For Western analyses, cell cultures were lysed in RIPA buffer (150 mM NaCl, 1% NP-40, 0.5% Na-deoxycholate, 0.1% SDS, 50 mM Tris, pH 7.4) supplemented with broad spectrum protease inhibitor cocktail (Sigma-Aldrich, St. Louis, MO). Protein concentration was determined using the Pierce bicinchoninic acid (BCA) Protein Assay Kit (ThermoFisher Scientific). Protein samples (15 μg) were electrophoresed on 8% SDS-acrylamide gels under denaturing conditions, then electroblotted onto nitrocellulose membranes. Membranes were blocked overnight in 5% BSA in TBST, washed and incubated overnight at 4 °C in TBST containing 5% BSA and primary antibody (diluted 1:1000). The following day, membranes were washed and incubated for 2 hours with corresponding IRDye infrared secondary antibody (diluted 1:10,000) (LI-COR Biosciences) in 2.5% milk. Proteins were visualized using an Odyssey Infrared Imaging System (LI-COR Biosciences, Lincoln, NE) and quantified using Odyssey 3.0.3 software and normalized to β-actin levels (ACTB). The primary antibodies used were mouse anti-human LMNA/C [JoL2] (Abcam, Cambridge, UK) and rabbit anti-beta-Actin [D6A8] (Cell Signaling Technology, Danvers, MA).

To quantify circulating markers of bone formation and turnover, blood was collected from six month-old mice at the time of sacrifice. Plasma was separated by centrifugation at 1500 g for 15 minutes. Levels of murine type I procollagen N-propeptide (PINP), tartrate-resistant acid phosphatase (TRAcP5b), and type I collagen C-telopeptide (CTX-I) were measured using Rat/Mouse PINP EIA and ELISA kits (Immunodiagnostic Systems), respectively. Additional markers tested by ELISA were mouse osteocalcin (OCN, ThermoFisher Scientific), type IIA N-propeptide (PIIANP, MyBioSource), and crosslinked type II C-telopeptide (CTX-II, Fisher Scientific).

**Immunofluorescence Microscopy.** Immunofluorescence imaging of bone cell populations *in vivo* was performed according to a modified procedure published by Dobson *et al* (Dobson *et al.*, 2016). The right femora were dissected from six month-old mice and fixed in 4% paraformaldehyde in PBS at 4 °C for two days. Bones were demineralized for three weeks in 14% EDTA at 4 °C under gentle shaking. Following dehydration, samples were paraffin-embedded and 5 µm bone tissue sections were cut and mounted on glass slides using standard protocols (Histoserv Inc). Slides were immersed in xylene for 20 minutes to remove paraffin, rehydrated in successive washes with 100%, 95%, and 70% EtOH for five minutes each, then incubated in 70% EtOH with 0.25% ammonium hydroxide for 1 hour to reduce autofluorescence derived from heme groups. Slides were then washed in 50% EtOH for ten minutes, fully rehydrated in tris-buffered saline (TBS) for five minutes and incubated at 80 °C in Dako target retrieval solution (Agilent) for 30 minutes. Slides were washed and blocked overnight at 4 °C in 4% mouse-on-mouse blocking solution (Vector Labs), 10% normal donkey serum, and 1% BSA in TBS. The next day slides were once again washed in TBS and treated with avidin and biotin blocking solutions for 15 minutes each. Primary antibodies were diluted 1:50 in 2% donkey serum, 1% BSA in TBS and applied to slides for 12 hours at 4°C. The primary antibodies used were mouse anti-LMNA/C [4c11] (Cell Signaling Technologies) and rabbit anti-progerin [Y288] antibody, a generous gift from K. Djabali, Technical University of Munich (McClintock *et al.*, 2006). The secondary antibodies AlexaFluor 594 donkey anti-rabbit and AlexaFluor 488 donkey anti-mouse (ThermoFisher Scientific) were diluted 1:200 in 2% donkey serum, 1% BSA in TBS and applied to the slides for 1 hour at room temperature. After washing in TBS for 15 minutes slides were mounted with Vectashield anti-fade mounting medium containing DAPI.

Confocal images were collected using a Zeiss LSM 880 confocal system (Airyscan, Carl Zeiss Inc.), mounted on an inverted microscope with a Plan-Apochromat 20x/0.75 or a Plan-Apochromat 63x/1.40 oil objective lens (Zeiss Axio Observer Z1). Bone tissues were placed on a coverglass-bottom dish (product #P35G-1.5-20-C, MatTek, Ashland, MA) where the number of z-sections needed to capture the entire bone tissue was determined to be a range from 6-22um in thickness. All images were acquired in LSM 880 mode using two PMTs and a 32-channel GaAsP-PMT detector. Excitation wavelengths of 405nm (0.8%), 488nm (1.5%), and 568nm (7%) were used for the detection of DAPI, A-type lamins ([4c11]), and progerin ([Y288]), respectively. All confocal images were 1,024 by 1,024 pixels, 16-bit, with a scan zoom of two and line averaged eight times. A software package was used for collection and post-processing of the images (Zeiss ZEN 2.3 (black)). Nuclear shape analysis was performed using a custom-written MATLAB program to measure nuclear morphology in cell cultures (Driscoll *et al.*, 2012).

**Histology.** Standard histology was performed on the right femora processed as described for immunofluorescence microscopy. Hematoxylin and eosin (H&E) and Safranin-O staining were performed according to standard procedures (Histoserv Inc., Germantown, MD). Tissue sections were imaged on a slide scanner with 20x objective (Zeiss AxioScan Z.1) and processed using image analysis software (Zen Blue, Carl Zeiss). Osteocyte lacunae counts were performed on 2 mm sections of mid-diaphyseal femora as previously described (Blouin *et al.*, 2024; Schmidt *et al.*, 2012). Lacunae were distinguished as those containing live cells with well-defined nuclei, or empty lacunae containing no nuclei, by blinded observers.

**MicroCT Analyses.** Left femora were dissected and cleaned of soft tissue, leaving the epiphyses intact. Sequential transaxial images were obtained through the bone cross-section using microcomputed tomography (µCT) (µCT40, Scanco Medical, Brütisellen, Switzerland). The samples were scanned using an integration time of 200 ms and a tube voltage and current of 70 kV and 114 mA, respectively. In the femora, regions of interest (ROI) were created for both cortical and trabecular bone regions. The trabecular ROI was located proximal to the distal femoral growth plate and extended 150 slices upward towards the proximal end. The diaphyseal cortical ROI started on the 55-percentile femur height from the proximal end, spanning 50 slices towards the distal end. Native Scanco software was used to measure total area, bone area, marrow area, cortical thickness, endosteal perimeter, periosteal perimeter, anterior-posterior bending moment of inertia (MOI), and tissue mineral density (TMD). Bone volume fraction (BV/TV, %), bone surface density (BS/BV, mm^−1^), connectivity density (ConnD, mm^−3^), Structure Model Index (SMI), trabecular number (Tb. N, mm^−1^), trabecular thickness (Tb. Th, mm), trabecular spacing (Tb. Sp, mm), apparent and mineral densities, and degree of anisotropy (DA) were calculated for trabecular bone ROIs. Images were calibrated using manufacturer-supplied hydroxyapatite phantoms to convert X-ray attenuation to a known mineral density.

**Mechanical Testing.** Femora were loaded to failure in three-point bending as previously described (Cubria *et al.*, 2020). Testing was performed using a load frame under displacement control conditions (MTS200 Synergy, MTS Inc., Eden Prairie, MN) with an 8mm support span. Specimens were thawed to room temperature, rehydrated for 30 min, and then loaded to failure at an axial strain rate of 0.01 mm/s. An in-house-developed program (MATLAB, MathWorks, Natick, MA) was used to analyze the raw force-displacement data and to calculate all three-point bending parameters.

**Statistical Analyses.** Unless otherwise stated, significant differences were determined by Welch’s t-test assuming unequal variances.

**Supplemental Data**

**Table S1. Transgene correction at 6 months following ABE treatment at 1 and 4 months.**

| **Mouse ID** |  | **Genotype** |  | **Age of Treatment** |  | **% c.1824T>C Edit** |
| --- | --- | --- | --- | --- | --- | --- |
|  |  |  |  |  |  |  |
|  |  |  |  |  |  |  |
| F118500 |  | *LMNA*^G/G^ |  | 1M |  | 1.58 |
| F218501 |  | *LMNA*^G/G^ |  | 1M |  | 18.69 |
| M418514 |  | *LMNA*^G/G^ |  | 1M |  | 6.70 |
| M2+618525 |  | *LMNA*^G/G^ |  | 1M |  | 11.87 |
| M218531 |  | *LMNA*^G/G^ |  | 1M |  | 10.54 |
|  |  |  |  |  |  |  |
| avg |  |  |  |  |  | 9.88 |
| sd |  |  |  |  |  | 6.34 |
|  |  |  |  |  |  |  |
|  |  |  |  |  |  |  |
| F118183 |  | *LMNA*^G/G^ |  | 4M |  | 1.15 |
| F118197 |  | *LMNA*^G/G^ |  | 4M |  | 0.33 |
| F418200 |  | *LMNA*^G/G^ |  | 4M |  | 0.54 |
| M218152 |  | *LMNA*^G/G^ |  | 4M |  | 0.69 |
| M118145 |  | *LMNA*^G/G^ |  | 4M |  | 0.31 |
| M418176 |  | *LMNA*^G/G^ |  | 4M |  | 0.76 |
|  |  |  |  |  |  |  |
| avg |  |  |  |  |  | 0.63 |
| sd |  |  |  |  |  | 0.31 |
|  |  |  |  |  |  |  |

**Table S2. *Lmna*^+/+^, *LMNA*^G608G/+^ and *LMNA*^G608G/G608G^ Femoral Bone Structural Parameters**

|  |  | ***Lmna*^+/+^** | ***LMNA*^G608G/+^** | ***LMNA*^G608G/G608G^** | ***LMNA*^G608G/G608G^** | ***LMNA*^G608G/G608G^** |
| --- | --- | --- | --- | --- | --- | --- |
|  |  |  |  |  | **ABE P3** | **ABE P14** |
|  |  |  |  |  |  |  |
|  |  |  |  |  |  |  |
| Trabecular Bone Volume Fraction (BV/TV) | Males | 0.168 ± 0.045 | 0.159 ± 0.059^††^ | 0.076 ± 0.023^**^ | 0.094 ± 0.040^*^ | 0.087 ± 0.014^**^ |
|  | Females | 0.045 ± 0.017 | 0.059 ± 0.037 | 0.031 ± 0.007^*^ | 0.037 ± 0.004^†^ | 0.037 ± 0.007 |
|  | Combined | 0.110 ± 0.080 | 0.112 ± 0.076^††^ | 0.053 ± 0.029^**^ | 0.068 ± 0.041 | 0.064 ± 0.028^*^ |
|  |  |  |  |  |  |  |
| Trabecular Number (Tb N/mm) | Males | 4.277 ± 0.561 | 3.873 ± 0.905 | 3.828 ± 0.321^*^ | 3.930 ± 0.521 | 3.606 ± 0.335^**^ |
|  | Females | 2.834 ± 0.450 | 2.617 ± 0.276 | 2.540 ± 0.254 | 2.585 ± 0.281 | 2.982 ± 0.210^††^ |
|  | Combined | 3.593 ± 0.892 | 3.277 ± 0.927 | 3.184 ± 0.718 | 3.318 ± 0.813 | 3.323 ± 0.424 |
|  |  |  |  |  |  |  |
| Trabecular Thickness (Tb Th, mm) | Males | 0.059 ± 0.008 | 0.061 ± 0.006^†††^ | 0.042 ± 0.003^***^ | 0.048 ± 0.003^**††^ | 0.050 ± 0.003^**†††^ |
|  | Females | 0.043 ± 0.004 | 0.047 ± 0.007^††^ | 0.037 ± 0.002^***^ | 0.046 ± 0.003^†††^ | 0.041 ± 0.003^†^ |
|  | Combined | 0.051 ± 0.010 | 0.054 ± 0.010^†††^ | 0.039 ± 0.004^***^ | 0.047 ± 0.003^†††^ | 0.046 ± 0.005^*††^ |
|  |  |  |  |  |  |  |
| Trabecular Mineral Density (Tb TMD, mg/cm^3^) | Males | 204.67 ± 55.87 | 195.06 ± 56.34^††^ | 118.10 ± 24.07^***^ | 126.20 ± 38.69^**^ | 132.85 ± 18.92^**^ |
|  | Females | 105.05 ± 16.98 | 112.79 ± 27.49^†^ | 82.76 ± 10.84^**^ | 92.28 ± 19.97 | 83.09 ± 8.69^**^ |
|  | Combined | 157.48 ± 65.58 | 156.08 ± 60.86^††^ | 100.43 ± 25.67^**^ | 110.79 ± 34.96^*^ | 110.24 ± 29.74^*^ |
|  |  |  |  |  |  |  |
| Structural Model Index (SMI) | Males | 1.988 ± 0.670 | 1.940 ± 0.734^††^ | 2.746 ± 0.335^**^ | 2.801 ± 0.543^*^ | 2.740 ± 0.184^**^ |
|  | Females | 3.094 ± 0.167 | 2.563 ±0.617^*^ | 2.992 ± 0.183 | 3.123 ± 0.126 | 3.289 ± 0.368 |
|  | Combined | 2.511 ± 0.747 | 2.235 ± 0.735^††^ | 2.869 ± 0.291 | 2.947 ± 0.427 | 2.989 ± 0.392* |
|  |  |  |  |  |  |  |
| Degree of Anisotropy (DA) | Males | 1.369 ± 0.141 | 1.444 ± 0.161^††^ | 1.240 ± 0.044^*^ | 1.244 ± 0.128 | 1.235 ± 0.042^*^ |
|  | Females | 1.222 ± 0.050 | 1.173 ± 0.074 | 1.194 ± 0.055 | 1.167 ± 0.046 | 1.174 ± 0.062 |
|  | Combined | 1.299 ± 0.129 | 1.315 ± 0.187^†^ | 1.217 ± 0.054^*^ | 1.209 ± 0.103^*^ | 1.207 ± 0.059^*^ |
|  |  |  |  |  |  |  |
|  |  |  |  |  |  |  |
| Cortical Tissue Mineral Density (Ct TMD, mg/cm^3^) | Males | 1210.94 ± 9.59 | 1206.90 ± 19.52 | 1211.24 ± 18.59 | 1211.13 ± 14.16 | 1203.90 ± 15.45 |
|  | Females | 1253.47 ± 22.91 | 1270.60 ± 32.08^†^ | 1245..01 ± 17.19 | 1253.02 ± 6.72 | 1258.45 ± 17.22 |
|  | Combined | 1232.20 ± 27.71 | 1238.75 ± 41.66 | 1228.92 ± 24.53 | 1232.08 ± 24.29 | 1231.17 ± 32.48 |
|  |  |  |  |  |  |  |
| Cortical Area (Ct Ar, mm^2^) | Males | 0.864 ± 0.095 | 0.884 ± 0.079^†††^ | 0.638 ± 0.055^***^ | 0.668 ± 0.041^***^ | 0.691 ± 0.054^***^ |
|  | Females | 0.798 ± 0.025 | 0.814 ± 0.054^†††^ | 0.620 ± 0.032^***^ | 0.642 ± 0.025^***^ | 0.663 ± 0.071^**^ |
|  | Combined | 0.831 ± 0.076 | 0.849 ± 0.075^†††^ | 0.628 ± 0.044^***^ | 0.655 ± 0.035^***^ | 0.677 ± 0.062^***†^ |
|  |  |  |  |  |  |  |
| Cortical Cross-sectional Thickness (Ct Th, mm) | Males | 0.171 ± 0.009 | 0.177 ± 0.012^†††^ | 0.155 ± 0.012^**^ | 0.163 ± 0.011 | 0.167 ± 0.005^†^ |
|  | Females | 0.189 ± 0.008 | 0.194 ± 0.017^†††^ | 0.165 ± 0.007^***^ | 0.174 ± 0.010^*^ | 0.181 ± 0.015^†^ |
|  | Combined | 0.180 ± 0.012 | 0.185 ± 0.017^†††^ | 0.160 ± 0.10^***^ | 0.168 ± 0.011^*†^ | 0.174 ± 0.013^††^ |
|  |  |  |  |  |  |  |
| Cortical Porosity (Ct Po) | Males | 0.271 ± 0.028 | 0.274 ± 0.054 | 0.346 ± 0.115 | 0.293 ± 0.043 | 0.295 ± 0.049 |
|  | Females | 0.287 ± 0.060 | 0.282 ± 0.051 | 0.276 ± 0.028 | 0.269 ± 0.043 | 0.259 ± 0.018 |
|  | Combined | 0.279 ± 0.046 | 0.278 ± 0.051 | 0.309 ± 0.087 | 0.281 ± 0.042 | 0.277 ± 0.040 |
|  |  |  |  |  |  |  |
| Mean Marrow Area (Ma Ar, mm^2^) | Males | 1.278 ± 0.150 | 1.331 ± 0.156^†††^ | 0.940 ± 0.156^***^ | 0.891 ± 0.044^***^ | 0.838 ±0.082^***^ |
|  | Females | 0.929 ± 0.081 | 0.945 ± 0.088^†††^ | 0.775 ± 0.059^***^ | 0.710 ± 0.065^***^ | 0.667 ± 0.036^***†††^ |
|  | Combined | 1.103 ± 0.213 | 1.138 ± 0.234^†††^ | 0.853 ± 0.141^***^ | 0.800 ± 0.109^***^ | 0.753 ± 0.108^***†^ |
|  |  |  |  |  |  |  |
| Cortical Moment of Inertia (MOI, mm^4^) | Males | 0.498 ± 0.099 | 0.519 ± 0.082^†††^ | 0.268 ± 0.052^***^ | 0.272 ± 0.029^***^ | 0.273 ± 0.041^***^ |
|  | Females | 0.352 ± 0.033 | 0.362 ± 0.035^†††^ | 0.220 ± 0.022^***^ | 0.216 ± 0.014^***^ | 0.219 ± 0.038^***^ |
|  | Combined | 0.425 ± 0.104 | 0.440 ± 0.101^†††^ | 0.243 ± 0.046^***^ | 0.244 ± 0.036^***^ | 0.246 ± 0.047^***^ |
|  |  |  |  |  |  |  |

Males, 6 months, *Lmna*^+/+^ n=10, *LMNA*^G/+^ n=10, *LMNA*^G/G^ n=10, *LMNA*^G/G^ P3 n=6, *LMNA*^G/G^ P14 n=6

Females, 6 months, *Lmna*^+/+^ n=9, *LMNA*^G/+^ n=9, *LMNA*^G/G^ n=10, *LMNA*^G/G^ P3 n=6, *LMNA*^G/G^ P14 n=6

*p < 0.05, **p < 0.01, ***p < 0.001 versus *Lmna*^+/+^ littermates

^†^p < 0.05, ^††^p < 0.01, ^†††^p < 0.001 versus untreated *LMNA*^G/G^ littermates

**Table S3. *Lmna*^+/+^, *LMNA*^G608G/+^ and *LMNA*^G608G/G608G^ Femoral Bone Physical Parameters**

|  |  | ***Lmna*^+/+^** | ***LMNA*^G608G/+^** | ***LMNA*^G608G/G608G^** | ***LMNA*^G608G/G608G^** | ***LMNA*^G608G/G608G^** |
| --- | --- | --- | --- | --- | --- | --- |
|  |  |  |  |  | **ABE P3** | **ABE P14** |
|  |  |  |  |  |  |  |
|  |  |  |  |  |  |  |
| Elastic Properties |  |  |  |  |  |  |
|  |  |  |  |  |  |  |
| Yield Load (N) | Males | 11.71 ± 2.61 | 10.95 ± 2.73^††^ | 7.68 ± 1.90^**^ | 11.76 ± 1.44^†††^ | 11.80 ± 1.81^††^ |
|  | Females | 10.18 ± 1.24 | 10.59 ± 2.19 | 8.79 ± 2.22 | 9.88 ± 2.40 | 10.39 ± 3.09 |
|  | Combined | 10.94 ± 2.14 | 10.77 ± 2.41^††^ | 8.29 ± 2.11^***^ | 10.82 ± 2.13^††^ | 11.09 ± 2.52^††^ |
|  |  |  |  |  |  |  |
| Yield Displacement (mm) | Males | 0.11 ± 0.03 | 0.11 ± 0.04 | 0.12 ± 0.04 | 0.13 ± 0.02 | 0.16 ± 0.06 |
|  | Females | 0.11 ± 0.04 | 0.11 ± 0.04 | 0.14 ± 0.06 | 0.12 ± 0.03 | 0.12 ± 0.05 |
|  | Combined | 0.11 ± 0.03 | 0.11 ± 0.04 | 0.13 ± 0.05 | 0.13 ± 0.02 | 0.14 ± 0.05 |
|  |  |  |  |  |  |  |
| Stiffness (N/mm) | Males | 109.69 ± 27.87 | 102.71 ± 20.81^†††^ | 69.14 ± 9.85^**^ | 81.60 ± 17.41^*^ | 82.19 ± 10.63^*†^ |
|  | Females | 109.47 ± 26.64 | 111.58 ± 24.09^††^ | 77.82 ± 22.14^**^ | 88.96 ± 24.54 | 91.30 ± 23.28 |
|  | Combined | 109.58 ± 26.53 | 107.15 ± 22.37^†††^ | 73.91 ± 17.85^***^ | 85.28 ± 20.64^**^ | 86.74 ± 17.90^**^ |
|  |  |  |  |  |  |  |
| Plastic Properties |  |  |  |  |  |  |
|  |  |  |  |  |  |  |
| Fracture Load (N) | Males | 16.08 ± 4.05 | 15.92 ± 4.18 ^†^ | 11.63 ± 2.82 * | 9.73 ± 2.85 ** | 13.80 ± 4.79 |
|  | Females | 14.51 ± 4.58 | 18.07 ± 5.30 ^††^ | 11.85 ± 3.53 | 11.60 ± 1.68 | 15.03 ± 3.64 |
|  | Combined | 15.30 ± 4.29 | 16.99 ± 4.78 ^†††^ | 11.75 ± 3.15 ** | 10.66 ± 2.43 ** | 14.41 ± 4.10^†^ |
|  |  |  |  |  |  |  |
| Post-Yield Displacement (PYD, mm) | Males | 0.42 ± 0.29 | 0.65 ± 0.50 | 0.35 ± 0.22 | 0.59 ± 0.29 | 0.36 ± 0.25 |
|  | Females | 0.54 ± 0.27 | 0.45 ± 0.20 | 0.48 ± 0.34 | 0.45 ± 0.11 | 0.32 ± 0.25 |
|  | Combined | 0.48 ± 0.28 | 0.55 ± 0.38 | 0.42 ± 0.29 | 0.52 ± 0.22 | 0.34 ± 0.24 |
|  |  |  |  |  |  |  |
| Total Properties |  |  |  |  |  |  |
|  |  |  |  |  |  |  |
| Maximum Load (N) | Males | 19.25 ± 2.42 | 19.97 ± 2.60^†††^ | 13.47 ± 2.28^***^ | 17.05 ± 1.59^*††^ | 17.55 ± 1.88^††^ |
|  | Females | 19.64 ± 1.75 | 21.01 ± 3.16^†††^ | 14.92 ± 1.80^***^ | 16.20 ± 1.76^**^ | 17.01 ± 2.05^*^ |
|  | Combined | 19.45 ± 2.07 | 20.49 ± 2.87^†††^ | 14.27 ± 2.11^***^ | 16.62 ± 1.66^***††^ | 17.28 ± 1.90^**†††^ |
|  |  |  |  |  |  |  |

Males, 6 months, *Lmna*^+/+^ n=10, *LMNA*^G/+^ n=10, *LMNA*^G/G^ n=10, *LMNA*^G/G^ P3 n=6, *LMNA*^G/G^ P14 n=6

Females, 6 months, *Lmna*^+/+^ n=9, *LMNA*^G/+^ n=9, *LMNA*^G/G^ n=10, *LMNA*^G/G^ P3 n=6, *LMNA*^G/G^ P14 n=6

*p < 0.05, **p < 0.01, ***p < 0.001 versus *Lmna*^+/+^ littermates

^†^p < 0.05, ^††^p < 0.01, ^†††^p < 0.001 versus untreated *LMNA*^G/G^ littermates

**Figure S1. Confocal immunofluorescence microscopy shows no detectable progerin in ABE-corrected *LMNA*^G/G^ osteoblasts *in vitro*.** Cells were imaged following treatment of wild-type (+/+) and *LMNA*^G/G^ (G/G) osteoblasts with saline, or *LMNA*^G/G^ osteoblasts treated with ABE (G/G ABE). Staining was performed with DAPI to visualize nuclei, phalloidin to visualize cytoplasmic area, and progerin-specific antibody. Nuclear blebbing is evident in untreated *LMNA*^G/G^, but not in wild-type or ABE-treated cells. Scale bar = 20µm.

**Figure S2. Quantitation of gene correction in *LMNA*^G/G^ osteoblasts *in vitro*.** (**A**) Left, Validation of droplet digital PCR SNP assay for *LMNA* c.1824C>T allele quantitation. A standard curve was generated using sequential dilutions of normal human and double copy murine transgenic fibroblast genomic DNA. Right, quantitation of C and T alleles in osteoblast cultures treated at a range of increasing MOI. NT, no treatment. (**B**) Quantitative RT-PCR results of transgene-derived *LMNA* transcript copy number (left) and specific transcripts expressed as a fraction of total transgene-derived *LMNA* transcripts (right). Transgene correction levels are denoted in red.(**C**) Quantitation of transgene-derived protein products based on Western analysis results. Levels of LMNA (A), progerin (P) and LMNC (C) are expressed as a fraction of total transgene-derived A-type lamins (left) or as a ratio relative to LMNC (right). *, p < 0.05; **, p < 0.01; ***, p < 0.001.

**Figure S3. Femoral growth plate histology of female mice.** Growth plates were stained with Safranin-O/Fast Green to identify cartilage proteoglycans (red) and mineralized bone tissue (green). Epiphysis and metaphysis are located to the left and right, respectively, of the growth plate. The cartilage to bone transitional zone is observed as red-stained (Safranin-O positive) protrusions intruding into the ossified (green) tissue. Scale bar = 100 μm.

**Figure S4. Femoral growth plate histology of male mice.** Growth plates were stained with Safranin-O/Fast Green to identify cartilage proteoglycans (red) and mineralized bone tissue (green). More intense Safranin-O staining is observed in the hypertrophic region of growth plates of untreated male *LMNA*^G/G^ mice. Scale bar = 100 μm.

**Figure S5. Adenine Base Editor expression remains active in bone at six months post-treatment.** (**A**) Gene expression analysis of RNA sequencing reads confirms continued expression of the dual vector ABE7.10 base editor system *in vivo* at six months of age in P14-treated murine bone tissue. The C-terminal portion of the dual vector ABE system expresses 2.85 ± 0.31 -fold more than the N-terminal fragment at this age. (**B**) Quantitation of transcripts from bulk RNA sequencing data showing increased *LMNA* (p < 0.02) and decreased progerin (p < 0.001) expression in bone tissue of P14-treated mice at six months of age. Saline, *LMNA*^G/G^ mice injected at P14 with saline only; ABE, *LMNA*^G/G^ mice treated at P14 with ABE.

**Supplemental References**

Bakker, A. D., and Klein-Nulend, J. 2012. Osteoblast isolation from murine calvaria and long bones. In Helfrich, M. and Ralston, S. (eds), *Bone Research Protocols*, 816, pp. 19-29. Humana Press, Totowa, NJ. <https://doi.org/10.1007/978-1-61779-415-5_2>.

Blouin, S., Hartmann, M. A., Fratzl-Zelman, N., Messmer, P., Whisenant, D., Erdos, M. R., et al. 2024. “Normal Bone Matrix Mineralization but Altered Growth Plate Morphology in the Lmna(G609G/G609G) Mouse Model of Progeria.” *Aging Dis,* 16, no. 5: 3204-3218. <https://doi.org/10.14336/AD.2024.1094>.

Cabral, W. A., Tavarez, U. L., Beeram, I., Yeritsyan, D., Boku, Y. D., Eckhaus, M. A., et al. 2021. “Genetic reduction of mTOR extends lifespan in a mouse model of Hutchinson-Gilford Progeria syndrome.” *Aging Cell,* 20, no. 9: e13457. <https://doi.org/10.1111/acel.13457>.

Cubria, M. B., Suarez, S., Masoudi, A., Oftadeh, R., Kamalapathy, P., DuBose, A., et al. 2020. “Evaluation of musculoskeletal phenotype of the G608G progeria mouse model with lonafarnib, pravastatin, and zoledronic acid as treatment groups.” *Proc Natl Acad Sci U S A,* 117, no. 22: 12029-12040. <https://doi.org/10.1073/pnas.1906713117>.

Dobin, A., Davis, C. A., Schlesinger, F., Drenkow, J., Zaleski, C., Jha, S., et al. 2013. “STAR: ultrafast universal RNA-seq aligner.” *Bioinformatics,* 29, no. 1: 15-21. <https://doi.org/10.1093/bioinformatics/bts635>.

Dobson, P. F., Rocha, M. C., Grady, J. P., Chrysostomou, A., Hipps, D., Watson, S., et al. 2016. “Unique quadruple immunofluorescence assay demonstrates mitochondrial respiratory chain dysfunction in osteoblasts of aged and PolgA(-/-) mice.” *Sci Rep,* 6: 31907. <https://doi.org/10.1038/srep31907>.

Driscoll, M. K., Albanese, J. L., Xiong, Z. M., Mailman, M., Losert, W., and Cao, K. 2012. “Automated image analysis of nuclear shape: what can we learn from a prematurely aged cell?” *Aging (Albany NY),* 4, no. 2: 119-132. <https://doi.org/10.18632/aging.100434>.

Gregory, C. A., Gunn, W. G., Peister, A., and Prockop, D. J. 2004. “An Alizarin red-based assay of mineralization by adherent cells in culture: comparison with cetylpyridinium chloride extraction.” *Anal Biochem,* 329, no. 1: 77-84. <https://doi.org/10.1016/j.ab.2004.02.002>.

Hartley, S. W., and Mullikin, J. C. 2015. “QoRTs: a comprehensive toolset for quality control and data processing of RNA-Seq experiments.” *BMC Bioinformatics,* 16, no. 1: 224. <https://doi.org/10.1186/s12859-015-0670-5>.

Koblan, L. W., Erdos, M. R., Wilson, C., Cabral, W. A., Levy, J. M., Xiong, Z. M., et al. 2021. “In vivo base editing rescues Hutchinson-Gilford progeria syndrome in mice.” *Nature,* 589, no. 7843: 608-614. <https://doi.org/10.1038/s41586-020-03086-7>.

Levy, J. M., Yeh, W. H., Pendse, N., Davis, J. R., Hennessey, E., Butcher, R., et al. 2020. “Cytosine and adenine base editing of the brain, liver, retina, heart and skeletal muscle of mice via adeno-associated viruses.” *Nat Biomed Eng,* 4, no. 1: 97-110. <https://doi.org/10.1038/s41551-019-0501-5>.

Li, H., and Durbin, R. 2009. “Fast and accurate short read alignment with Burrows-Wheeler transform.” *Bioinformatics,* 25, no. 14: 1754-1760. <https://doi.org/10.1093/bioinformatics/btp324>.

McClintock, D., Gordon, L. B., and Djabali, K. 2006. “Hutchinson-Gilford progeria mutant lamin A primarily targets human vascular cells as detected by an anti-Lamin A G608G antibody.” *Proc Natl Acad Sci U S A,* 103, no. 7: 2154-2159. <https://doi.org/10.1073/pnas.0511133103>.

Parfitt, A. M. 1977. “The cellular basis of bone turnover and bone loss: a rebuttal of the osteocytic resorption--bone flow theory.” *Clin Orthop Relat Res* no. 127: 236-247. Retrieved from <https://www.ncbi.nlm.nih.gov/pubmed/912987>

Schmidt, E., Nilsson, O., Koskela, A., Tuukkanen, J., Ohlsson, C., Rozell, B., et al. 2012. “Expression of the Hutchinson-Gilford progeria mutation during osteoblast development results in loss of osteocytes, irregular mineralization, and poor biomechanical properties.” *J Biol Chem,* 287, no. 40: 33512-33522. <https://doi.org/10.1074/jbc.M112.366450>.

Varga, R., Eriksson, M., Erdos, M. R., Olive, M., Harten, I., Kolodgie, F., et al. 2006. “Progressive vascular smooth muscle cell defects in a mouse model of Hutchinson-Gilford progeria syndrome.” *Proc Natl Acad Sci U S A,* 103, no. 9: 3250-3255. <https://doi.org/10.1073/pnas.0600012103>.

Wang, Y., Bergelson, S., and Feschenko, M. 2018. “Determination of Lentiviral Infectious Titer by a Novel Droplet Digital PCR Method.” *Hum Gene Ther Methods,* 29, no. 2: 96-103. <https://doi.org/10.1089/hgtb.2017.198>.
